# The absence of reporting standards and a lack of objective, performance-based outcomes following intramedullary nailing of tibial shaft fractures: findings from a scoping review into 179 articles

**DOI:** 10.1007/s00068-023-02338-1

**Published:** 2023-08-09

**Authors:** Simon Thwaites, John Abrahams, Dominic Thewlis, Mark Rickman

**Affiliations:** 1 Centre for Orthopaedic and Trauma Research, Adelaide Medical School, The University of Adelaide, Adelaide, South Australia Australia; 2 Department of Orthopaedics and Trauma, Royal Adelaide Hospital, Adelaide, South Australia Australia

**Keywords:** Infrapatellar, Suprapatellar, Tibial nailing, Anterior knee pain, Orthopaedics

## Abstract

**Purpose:**

This scoping review was conducted to summarise the outcome tools reported in the assessment of tibial shaft fractures treated with intramedullary (IM) nailing, with a key focus on knee pain and function, and performance-based outcomes.

**Methods:**

PubMed and Embase databases were searched on May 31, 2023. All study designs and populations were included, including ex vivo studies without fracture. Studies with only open or intra-articular fractures, or other fracture fixation, were excluded. Reported outcome tools and pertinent study characteristics were extracted and summarised.

**Results:**

Of 488 articles identified, 179 met the inclusion criteria. For in vivo studies (*n* = 152), there were 13,705 fractures; the IM nailing approach not described for 30% of these. There were 133 unique patient outcomes, with a binary assessment of knee pain (29% of studies) and Lysholm score (21%) most common. Only 10/152 (7%) in vivo studies included an objective, performance-based measure of knee function. Fracture union was most frequent (52%) of 81 different clinical outcomes. For ex vivo studies (*n* = 29), there were 408 tibias included, with nail insertion location most prevalent (66% of studies) of 34 reported outcomes.

**Conclusion:**

The heterogeneity of outcome tools reported limits comparison between studies and the most commonly reported patient outcomes may not be the most appropriate. Future studies should report the IM nailing approach and consider capturing both patient-reported and performance-based outcomes to help inform surgical decision making.

**Supplementary Information:**

The online version contains supplementary material available at 10.1007/s00068-023-02338-1.

## Introduction

Intramedullary (IM) nailing is the typical method of fixation for adult tibial shaft fractures, the most common long bone fracture [1]. Advantages of IM nailing include stable fixation with minimal soft-tissue dissection [2], high union rates [3], and early post-operative mobilisation [4]. Techniques to perform IM nailing vary, generally categorised by the nail insertion approach. Traditionally, infrapatellar nailing (IPN) is performed with the knee flexed and a transtendinous or medial/lateral paratendinous incision. Techniques with the knee in semi-extension were later developed [5] to reduce the risk of valgus and procurvatum deformities associated with proximal-third tibial shaft fractures [6]. In the last two decades, suprapatellar nailing (SPN), an intra-articular approach with the knee in semi-extension and utilising a quadriceps incision, has gained popularity.

There is a growing body of research, of varying study designs, investigating numerous clinical and patient outcomes following different IM nailing approaches. There are promising results for SPN, showing good alignment rates [7, 8], reduced fluoroscopy time [9–11], and improved nail entry point [12] and insertion angles [13]. However, there are conflicting results concerning knee pain and function [7, 9, 12]. Long-term anterior knee pain (AKP) is a well-recognised disadvantage of IM nailing, occurring in 10% [14] to 86% [15] of cases, and a frequently reported adverse outcome. Indeed, one of the main attractions of SPN is moving the nail insertion site away from the patellar tendon, damage to which is thought to play a part in the aetiology of AKP [16].

Although tibial IM nailing is being extensively explored, and there are consistent efforts to conduct systematic reviews and meta-analyses, there remains no conclusive evidence to inform surgical decision making on the optimal technique [17]. Due to the lack of validated outcome measures in this cohort, such reviews [18] are inherently limited in their ability to conduct pooled analyses from the disparate outcomes reported in the included studies. Recently (2021), one review [18] comparing IPN versus SPN, which included 16 studies (five randomised controlled trials (RCTs) and 11 observational studies), suggested that SPN is associated with reduced post-operative visual analogue scale (VAS) pain scores and improved functional outcomes (based on Lysholm [19] scores). However, only five studies reported pain scores, and the authors were unable to perform a meta-analysis for VAS due to the different scales in use. Eight studies included appropriate data for function scores, with five different function scores used (4 × Lysholm, 1 × Hospital for Special Knee Surgery [20], 1 × Kujala [21], 1 × Symptoms Subset of the International Knee Documentation Committee [22], and 1 × Oxford [23] knee scores). The validity of the outcomes from such reviews is questionable, particularly regarding knee function, as there are inherently low numbers pooled for each function score. This is highlighted in another 2021 review [17], which, although including substantially more articles (67 articles, including 45 retrospective studies), was only able to calculate pooled estimates for: the Lysholm score, based on six studies (IPN numbers: 274; SPN numbers: 111); the Iowa knee score [24], from two studies (IPN numbers: 67; SPN numbers: 0); and the Kujala score, from two studies (IPN numbers: 99; SPN numbers: 0). Further, a recent review (2021) [25] into the quality of RCTs comparing infrapatellar and suprapatellar approaches rated the majority of RCTs as poor according to the Modified Coleman Methodology Score [26] and Revised Consolidated Standards of Reporting Trials Score [27], with weaknesses frequently identified regarding the reporting of statistical methods and outcome assessment tools.

The outcome measures that are currently in use, being designed for other pathologies (e.g., osteoarthritis, total knee replacements, etc.), may not be truly applicable. As described by Morris et al. [28], some of these tools, due to their regular use, appear to have almost defined a de facto standard, despite inadequate evidence to support their widespread adoption. Notably, there appears to be a paucity of studies including objective, performance-based measures of knee function. The importance of capturing both self-reported and performance-based measures is well-established in other areas of orthopaedic research [29]. However, for tibial nailing, clinicians are currently restricted to evidence syntheses of knee function that rely entirely on patients’ self-reported function, which may not truly describe patient recovery.

The true extent and prevalence of the different outcome measures in use following tibial nailing is currently not known, and such an investigation lends itself towards a scoping review approach. Therefore, the purpose of this scoping review is to present an overview of the outcome tools being used to investigate tibial shaft fractures treated with IM nailing. Specifically, the aims of the study were to: (1) detail pertinent study characteristics, including study design and IM nail insertion approach(es) used; (2) summarise the number of reported patient and clinical outcome tools in in vivo studies, with a particular focus on measures of knee pain and function, and the use of performance-based measures; and (3) summarise the outcome tools reported in ex vivo studies.

Understanding of these outcomes will grant the readership a holistic view of the methods currently being used to investigate different IM nailing approaches. Such findings may provide recommendations for future studies by revealing gaps in knowledge, and ultimately enhance decision-making for clinicians and researchers regarding the optimal tibial nailing technique.

## Methods

The research team adhered to published scoping review frameworks [30–33]. The protocol (objectives, inclusion criteria, methods, etc.) was determined a priori and is provided as supplementary material (Online Resource 1). The five-stage approach was as follows:

### Stage 1: Identify the research question

A broad research question was developed as many factors are known to affect outcomes in this cohort: “what is known about the studies investigating tibial shaft fractures treated with IM nailing, and what outcomes are reported?”.

### Stage 2: Identify relevant studies

Inclusion and exclusion criteria are summarised in Table 1. A three-step search strategy was conducted:

**Table 1:** Inclusion and exclusion criteria for article screening

Inclusion criteria	Exclusion criteria
Intramedullary nail fixation	Review articles
All suprapatellar and infrapatellar nailing approaches	Non-tibial shaft fracture
Tibial shaft fractures	Open fractures only
All populations	Other fracture fixation
All study designs (including cadaveric studies without presence of tibial shaft fracture)	Intra-articular fractures
Original work	Nail removal only presented
English	Technical notes / technique description without a case series presented

#### Step 1: Initial limited search

An initial limited search was conducted with a university librarian of PubMed and Embase databases.

#### Step 2: Identify key words and index terms

The Step 1 search enabled database-specific title, abstract and index/mesh heading terms to be identified. The generalised Boolean search operations were: (“Fracture Fixation, Intramedullary” OR “bone nails” OR “intramedullary nail*”) AND (“tibial fractures” OR “tibia* shaft” OR “tibia*” OR “fracture*”) AND (“suprapatellar” OR “infrapatellar OR “semiextended” OR “retropatellar” OR “medial parapatellar” OR “lateral parapatellar” OR “transtendinous”). Complete database-specific search terms are provided as supplementary material (Online Resource 2). PubMed and Embase databases were searched on May 31, 2023.

#### Step 3: Searching of references and citations

Additional studies were identified from searching the reference lists of included studies.

### Stage 3: Study selection

Article screening was performed with an online tool (Covidence™). Two reviewers (*S.T.* and *J.A., or S.T. and D.T.*) independently screened articles for relevant titles and abstracts, and resolved any conflicts after discussion. The same reviewers independently performed full-text screening, with any conflicts at this level resolved by another reviewer (*M.R.*).

### Stage 4: Charting the data

Data extraction was completed by one researcher (*S.T.*) using the online tool. *D.T.* extracted data for ≈10% (*n* = 17) of included articles in order to assess extraction accuracy by overall agreement and Cohen’s kappa (*k*). The data extraction template was developed a priori (see supplementary material Online Resource 1), with level of evidence added following discussion of the interim results after piloting the data extraction template on 10% of included studies. Formally assessing the quality of included studies (e.g., risk of bias) was outside the scope of this scoping review, nevertheless, the level of evidence, as reported by the journal or according to Marx et al. [34], can be readily extracted to give some indication into overall study quality. Final extracted data included: author, title, year, and country of origin of the publication; general nailing focus (e.g., IPN, SPN, IPN versus SPN, etc.); detailed nailing approach (e.g., IPN transtendinous, IPN medial parapatellar, etc.); study design; the number of fractures (in vivo studies) or number of tibias (ex vivo studies) included and their nailing approach; level of evidence; follow-up times; outcomes reported; whether the locking technique, fracture type/location, open/closed fracture distribution, reaming method, post-operative weight bearing regime, and mechanism of injury were described; and whether nail removal was included.

Patient outcomes were defined as outcomes requiring direct patient input (often completed by the patient), generally in the form of answering questions related to pain or function, or completing tasks (e.g., quadriceps strength assessment). Objective, performance-based measures are a subset of patient outcomes. Clinical outcomes were defined as anything identified by medical staff, e.g., union, alignment, and complications. Range of motion was classified as a clinical outcome to be consistent with previous reviews [28].

Complications were often reported as a grouped outcome, but were recorded as individual outcomes if uniquely presented. Similarly, hardware failure was often reported as a generic outcome, but in cases where locking bolt or nail failure was specified this was recorded. Instances of union and time to union were grouped. Synonymous nailing approach descriptions, e.g., IPN transtendinous and IPN transpatellar, were grouped. For VAS scores, the scale must have been specified, i.e., the scale range was not assumed from the results if not explicitly reported. Authors were contacted for any missing data.

### Stage 5: Collating, summarising, and reporting the results

Extracted data were summarised using R (version 4.1.1, R Core Team, Austria) and RStudio (version 1.4.1717, RStudio Team, USA). R scripts (https://github.com/simon-thwaites) were developed to: analyse publication temporal trends, determine level of agreement of extracted data, and summarise group counts, e.g., numbers of different nailing techniques, reported outcomes, etc. No measures of central tendency were performed. The results and their implications were discussed amongst the research team.

## Results

### Study characteristics

**Fig. 1:**
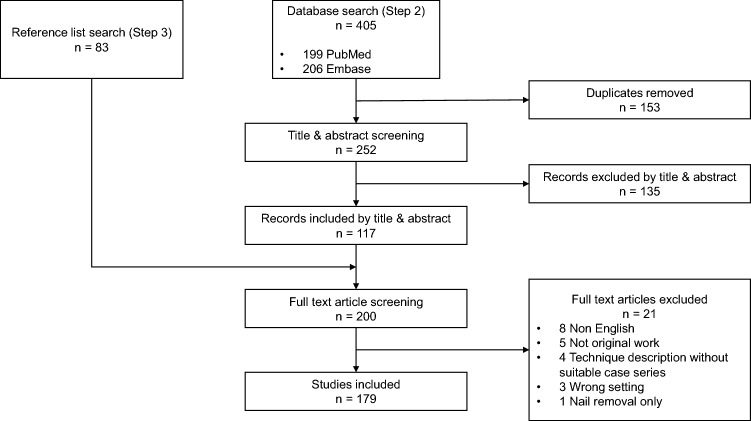
Preferred Reporting Items for Systematic Reviews and Meta-Analyses [30] flowchart

**Fig. 2:**
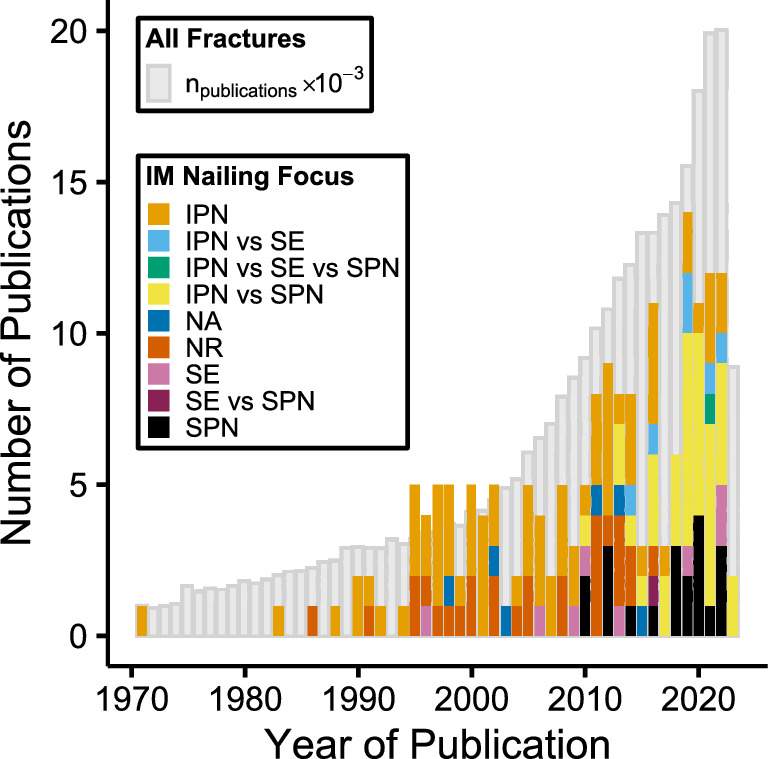
Number of publications by year grouped by intramedullary nailing focus. All “fracture” publications (${n}_{\mathrm{publications}}$) in PubMed also presented. *IM* intramedullary, *IPN* infrapatellar nailing, *SE* semi-extended, *SPN* suprapatellar nailing, *NA* not applicable, *NR* not reported

A total of 488 articles were identified yielding 179 papers for data extraction. The Preferred Reporting Items for Systematic Reviews and Meta-Analyses [30] flowchart is shown in Fig. 1. Data were extracted with 93.6% overall agreement, and mean *k*-value of 0.87 for individual items (range: 0.43–1.00). Ninety (50%) of the included tibial nailing articles were published within the last decade, showing a similar chronological publication trend to fractures in general (Fig. 2). Articles were identified from 27 different countries, with 60 (34%) originating from the USA (Table 2). There were 150 in vivo studies, and 27 ex vivo studies; two papers included both study types. Retrospective cohort studies were most prevalent (46%) (Table 3). Most studies (49%) were level III evidence (Online Resource 2).

**Table 2:** Number of intramedullary nailing publications (n (%)) by country of origin

Country	*n*	(%)
United States	60	(33.5)
Other	31	(17.3)
UK	24	(13.4)
China	14	(7.8)
Canada	12	(6.7)
Turkey	10	(5.6)
India	8	(4.5)
Germany	7	(3.9)
Finland	5	(2.8)
Austria	4	(2.2)
Italy	4	(2.2)

'Other' threshold < 4 included as supplementary material (Online Resource 2)

**Table 3:** Number (*n* (%)) of intramedullary nailing study designs

Study design	*n*	(%)
Retrospective cohort study	84	(46.4)
Cadaveric	29	(16.0)
Prospective cohort study	26	(14.4)
Case series	20	(11.0)
Randomised controlled trial	19	(10.5)
Case control study	2	(1.1)
Case report	1	(0.6)

### In vivo studies

Of the 152 in vivo studies: 10 studies reported only patient outcomes, 50 reported only clinical outcomes, and 92 reported both patient and clinical outcomes. The number of reported patient and clinical outcomes ranged from 1 to 23 per study. The total number of fractures included was 13,705 (range: 2–1226), with the medial parapatellar (20% of fractures) and transtendinous (18%) IPN approaches most common (Table 4). A substantial number of fractures (30%) did not have a specified nailing approach. One study [35] reported economic outcomes (Medical Consumption Questionnaire [36], Productivity Cost Questionnaire [37], and direct and indirect medical costs).

**Table 4:** Summary of intramedullary nailing insertion techniques

IM nailing approach	In vivo		Ex vivo		Totals	
	${n}_{fracs}$	(%)	${n}_{tibias}$	(%)	${n}_{total}$	(%)
*IP*						
Lateral parapatellar	10	(0.1)	37	(9.1)	47	(0.3)
Medial parapatellar	2774	(20.2)	128	(31.4)	2902	(20.9)
Transtendinous	2486	(18.1)	43	(10.5)	2529	(17.9)
Grouped transtendinous and lateral parapatellar	14	(0.1)	–	–	14	(0.1)
Grouped transtendinous and medial parapatellar	228	(1.7)	–	–	228	(1.6)
Grouped transtendinous and medial/lateral parapatellar	215	(1.6)	–	–	215	(1.5)
Unspecified	1105	(8.1)	–	–	1105	(7.8)
*IP*						
**Subtotal**	**6832**	**(49.9)**	**208**	**(51.0)**	**7040**	**(49.9)**
Lateral parapatellar (EA)	245	(1.8)	11	2.7	256	(1.8)
Lateral paratendinous (EA)	83	(0.6)	–	–	40	(0.6)
Medial parapatellar (EA)	51	(0.4)	5	1.2	56	(0.4)
Medial parapatellar (IA)	100	(0.7)	–	–	100	(0.7)
Superomedial (IA)	84	(0.6)	–	–	84	(0.6)
*SE*						
**Subtotal**	**563**	**(4.1)**	**16**	**3.9**	**536**	**(4.1)**
Chinese aircraft-shaped sleeve	33	(0.2)	–	–	33	(0.2)
Lateral	22	(0.2)	10	(2.5)	32	(0.2)
Midline	954	(7.0)	85	(20.8)	1019	(7.4)
Unspecified	1230	(9.0)	33	(8.1)	1221	(8.9)
*SP*						
**Subtotal**	**2239**	**(16.3)**	**128**	**(31.4)**	**2305**	**(16.8)**
*ND*						
**Subtotal**	**4071**	**(29.7)**	**56**	**(13.7)**	**4127**	**(29.2)**
**Total**	**13,705**	**(100)**	**408**	**(100)**	**13,756**	**(100)**

For in vivo studies, the number of tibial shaft fractures* (*${n}_{fracs}$); for ex vivo studies, the number of tibias *(*${n}_{tibias}$*)*

*IM* intramedullary, *IP* infrapatellar, *SE* semi-extended, *EA*, extra-articular, *IA* intra-articular, *SP* suprapatellar, *ND* no description

**Fig. 3:**
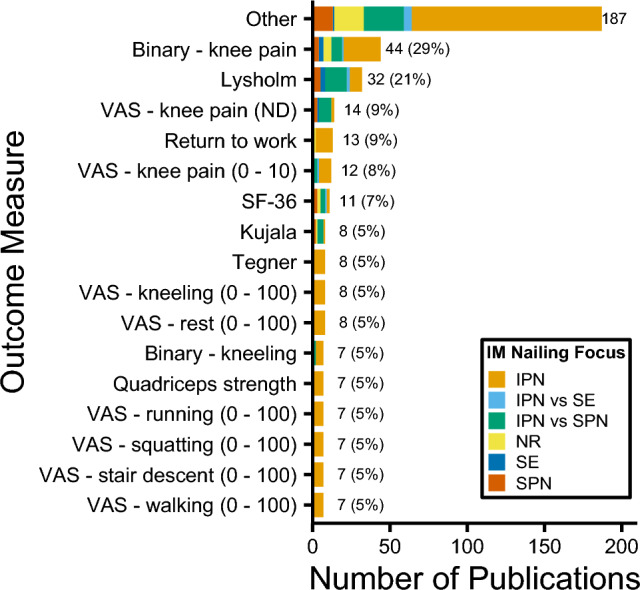
Patient outcomes for in vivo studies grouped by intramedullary nailing focus. 'Other' threshold < 7 included as supplementary material. *IM* intramedullary, *IPN* infrapatellar nailing, *SE* semi-extended, *SPN* suprapatellar nailing, *NR* not reported, *VAS* visual analogue scale; SF-36, 36-Item Short Form Health Survey

A total of 133 unique patient outcomes were identified (range: 1–15), with a binary (yes/no) assessment of knee pain (44/152 (29%) in vivo studies), Lysholm [19] score (32/152 (21%)), and a VAS score for knee pain with an undescribed scale (14/152 (9%)) were most commonly reported (Fig. 3). Combined, VAS (*n* = 24) and numerical rating scale (NRS; *n* = 38) scores, accounted for 62/133 (47%) unique patient outcomes, though VAS scores appeared in more in vivo studies than NRSs (36/152 (24%) versus 10/152 (7%)). The 24 different VAS outcomes included three different scales: [0–10], ﻿[1–10], [0–100]. There was no description of the scale in 14/36 (39%) studies that included a VAS score. The 38 unique NRS outcomes had five different scales: [0–3], [1–7], [0–10],﻿ ﻿[1–10]. The most common VAS and NRS domains were knee pain (included in 29/152 (19%) studies), kneeling (16/152 (11%)), walking (15/152 (10%)), and stair ascent/descent (14/152 (9%)).

Only 10/102 (10%) studies assessing patient outcomes (10/152 (7%) of all in vivo studies) reported a performance-based measure (Table 5). Seven studies reported quadriceps strength (7 /102 (7%)); six reported hamstrings strength (6/102 (6%)); and there were single instances (1/102 (1%)) of the six-minute walk test [38]; a poorly described kneeling test [39]; and a well-described described kneeling test, the Aberdeen Weight-Bearing Test (Knee) (AWT-K) [40]. The only prospective study comparing IPN and SPN that included an objective measure was the RCT by MacDonald et al. [40], utilising the AWT-K.

**Table 5:** Objective, performance-based outcome measures in use in in vivo studies

Author	Year	Country	Study design	IM nail focus	A1	${n}_{A1}$	A2	${n}_{A2}$	Objective outcome
Nyland [50]	2001	United States	Retrospective	IPN	IPN medial parapatellar	10	*NA*	–	Quadriceps strength, hamstrings strength
Toivanen [15]	2002	Finland	RCT	IPN	IPN transpatellar	21	IPN medial parapatellar	21	Quadriceps strength, hamstrings strength
Väistö [47]	2004	Finland	Prospective	IPN	IPN transpatellar	20	IPN medial parapatellar	20	Quadriceps strength, hamstrings strength
Väistö [49]	2007	Finland	Prospective	IPN	IPN transpatellar	14	IPN medial parapatellar	14	Quadriceps strength, hamstrings strength
Väistö [52]	2008	Finland	RCT	IPN	IPN transpatellar	14	IPN medial parapatellar	14	Quadriceps strength, hamstrings strength
Herren [39]	2012	Germany	Case series	SPN	SPN unspecified	26	*NA*	–	Kneeling test (ND)
Theriault [38]	2012	Canada	Prospective	IPN	IPN transpatellar	70	*NA*	–	Six-minute walk test
MacDonald [40]	2019	UK	RCT	IPN vs SPN	SPN unspecified	53	IPN medial parapatellar	42	Aberdeen Weight-Bearing Test (Knee)
Özbek [48]	2019	Turkey	Retrospective	IPN	IPN transpatellar	40	*NA*	–	Quadriceps strength, hamstrings strength
Stella [51]	2019	Italy	Prospective	SE	Semi-extended lateral parapatellar (EA)	70	*NA*	–	Quadriceps strength

*IM* intramedullary, *A1* approach 1, ${n}_{A1}$ number of A1 approach, A2 approach 2; ${n}_{A2}$ number of A2 approach, *IPN* infrapatellar nail, *RCT* randomised controlled trial, *SPN* suprapatellar nail, SE semi-extended, *EA* extraarticular, *NA* not applicable, *ND* not described

**Fig. 4:**
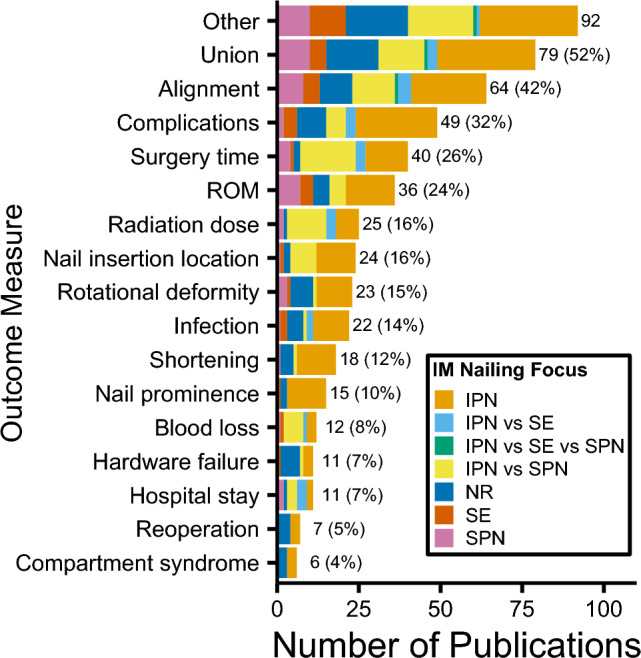
Clinical outcomes for in vivo studies grouped by intramedullary nailing focus. 'Other' threshold < 6 included as supplementary material. *IM* intramedullary, *IPN* infrapatellar nailing, *SE* semi-extended, *SPN* suprapatellar nailing, *NR* not reported, *ROM* range of motion

**Fig. 5:**
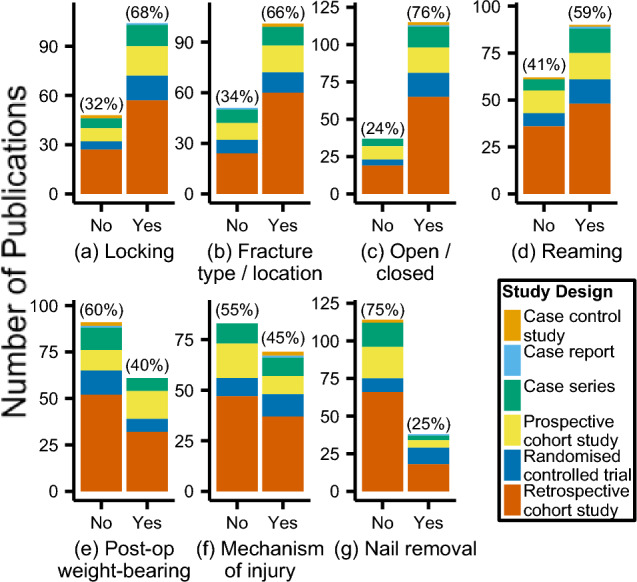
Distribution (no/yes, and percentage of studies) of whether in vivo studies provided a description of **a** locking technique, **b** fracture type/location, **c** open/closed fracture, **d** reaming technique, **e** post-operative weight-bearing regime, **f** mechanism of injury, and **g** if nail removal was included. Study characteristics are grouped by study design

There were 81 different clinical outcomes (range: 1–12): fracture union (79/152 (52%) in vivo studies), alignment (64/152 (42%)), and complications (49/152 (32%)) were most reported (Fig. 4). The most frequently reported follow-up times were six and 12 months (38/152 (25%) each) (Online Resource 2). The majority of studies provided a description of nail locking (104/152 (68%), Fig. 5a), fracture type/location (101/152 (66%), Fig. 5b), open/closed fracture distribution (115/152 (76%), Fig. 5c), and reaming (90/152 (59%), Fig. 5d); however, most did not describe the post-operative weight-bearing regime (91/152 (60%), Fig. 5e) or the mechanisms of injury (83/152 (55%), Fig. 5f). Nail removal was not included in most studies (114/152 (75%), Fig. 5g).

### Ex vivo studies

**Fig. 6:**
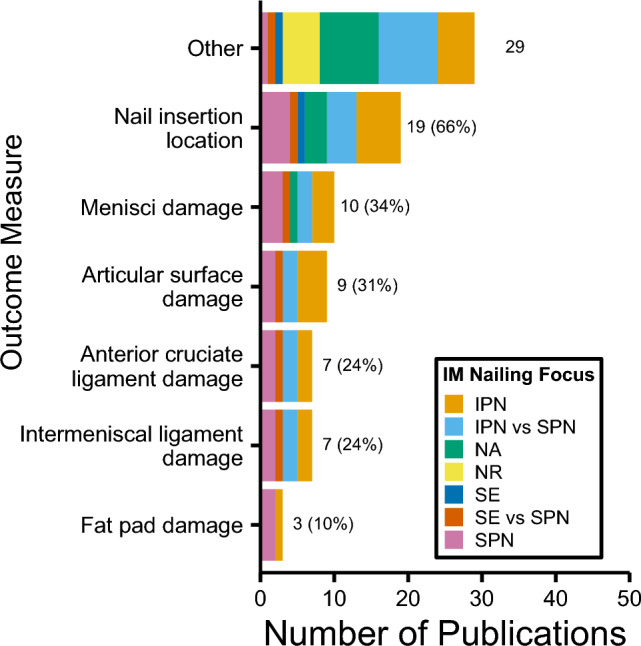
Outcomes for ex vivo studies grouped by intramedullary nailing focus. 'Other' threshold < 3 included as supplementary material. *IM* intramedullary, *IPN* infrapatellar nailing, *SE* semi-extended, *SPN* suprapatellar nailing, *NA* not applicable, *NR* not reported

For ex vivo studies (*n* = 29), there were 34 outcomes reported (range: 1–7 outcomes per study), with nail insertion location (66% of all ex vivo studies), menisci damage (34%), and articular surface damage (31%) most prevalent (Fig. 6). There were 408 tibias included (range: 1–57), with the medial parapatellar IPN (31% of tibias) and midline SPN approaches (21%) most common (Table 4). Full listings of reported outcomes are provided as supplementary material (Online Resource 3).

## Discussion

As expected, there was a high degree of heterogeneity in the outcomes reported. Surprisingly, perhaps the simplest outcome to capture, the presence of AKP, despite being most frequently reported, occurred in less than a third (29%) of in vivo studies. Of the 44/152 (29%) studies reporting AKP, only 10/44 (23%) studies reported an accompanying VAS or NRS score (8 and 2 studies, respectively). The use of binary measures alone is problematic as they are unable to distinguish differing degrees of pain, or pain only with certain activities; require large participant numbers for adequate study power [41]; and are limited in their ability to detect differences between treatments [42]. Conversely, continuous scales provide a more clinically informative statistic: they enable the calculation of the proportion of patients who achieve a clinically important improvement in pain scores [43].

The ability of VAS and NRS scores to granularize pain is clearly advantageous, and a likely reason for their popularity. These scores, accounting for almost half (47%) of all unique scores, were used to capture a host of different outcomes. However, the scales reported were inconsistent and often undescribed, with over a third (39%) of studies including a VAS outcome not defining the scale used. This discrepancy in VAS and NRS scales in the literature has hindered some meta-analyses in not being able to present pooled pain scores [18]. Future studies should ensure to report the scale used, and seek to conform to a [0–10] or [0–100] scale, as these are most common and are readily aggregated. Further, adopting notion to improve clarity in interpreting VAS and NRS scores, e.g., ${{VAS}_{AKP}}_{0}^{10}$, ${{VAS}_{walk}}_{0}^{10}$, would remove any ambiguity by succinctly detailing both the specific outcome and scale.

This scoping review builds on a previous study by Morris et al. [28] identifying the outcomes in use following tibial shaft fracture. Given the popularity of IM nailing to treat tibial shaft fractures, and the particular concerns regarding AKP and knee function following IM fixation, a more targeted scoping review was warranted. Though Morris et al. provides excellent detail into the clinical parameters reported following tibial shaft fracture, their review does not give a true indication into the varied nature of patient outcomes in use following IM nailing. Morris et al. describes 23 different clinician- and patient-reported patient outcomes from the 117 articles retrieved (grouping VAS and NRS scores), with six occurrences of the Lysholm score, and 9 occurrences of generic VAS/NRS scores for pain. Comparatively, this review describes 133 unique patient outcomes from 152 in vivo studies, with 32 studies using the Lysholm score, and 46 studies including a VAS/NRS score, and provides greater detail into the different scales and domains for VAS and NRS scores.

In addition to highlighting the heterogenous nature of outcomes reported following IM nailing, a major strength of this review is revealing the scarce use of objective, performance-based outcome measures. The importance of including performance measures has been emphasised in other areas, e.g., after total knee arthroplasty [29, 44, 45]. Anandasivam et al. [46] showed a bimodal age distribution of tibial shaft fracture patients, with peaks at 21 and 47 years of age; given this younger, generally more active and higher functioning cohort, performance-based measures may also play a valuable role in describing patient recovery after tibial nailing. Only 10 studies included a performance-based measure (7% of in vivo studies overall), four coming from the same group out of Finland. Hamstrings strength deficits have been shown to be related to AKP in transpatellar [47, 48] and medial parapatellar [47] IPN groups. Similarly, quadriceps strength deficits have also been shown to be related to AKP in transpatellar [49] and medial parapatellar [49, 50] IPN groups. Stella et al. [51] reported quadriceps strength following a semi-extended extraarticular lateral parapatellar approach, but there were no comparative studies found comparing semi-extended or SPN approaches with IPN approaches, which is surprising given permanent quadriceps deficit is a concern regarding SPN [6]. The only prospective study comparing SPN and IPN that included an objective measure was the RCT by MacDonald et al. [40], accounting for less than 1% (95/13,705) of patients in the literature. Macdonald et al. [40] used the AWT-K as an objective, surrogate measure for AKP. Their results showed the SPN group kneeling with a greater proportion of body weight transferred through the injured leg compared to the IPN group. It is unknown why so few studies have included performance-based outcomes in this cohort but shows a substantial gap in the literature, particularly regarding SPN versus IPN, which future studies should address. Further, readers should exercise caution in interpreting pooled function scores from current reviews given the gap in knowledge regarding objective, performance-based measures following IM nailing.

As there remains a need for at least one questionnaire to be validated for this cohort, the applicability of questionnaires such as the Lysholm score, and others frequently reported, remains questionable. These scores, being designed for other pathologies, may not be the most appropriate. In particular, the literature clearly indicates patients may have difficulty kneeling following IM nailing: up to 50% of patients are unable to kneel at all [53], it causes the most severe pain [17, 54], intensifies existing pain in 60% of cases [55], and has incidence reported as high as 92% [53]. A score comprising a kneeling component seems appropriate for this cohort. Yet, notably, none of the top-three reported composite pain/function scores (Lysholm (21%), Kujala (5%), and Tegner [56] (5%)) include a kneeling component. Given there may be a discrepancy in perceived versus actual kneeling ability, highlighted by Hassaballa et al. [57] showing up to 44% of patients incorrectly classifying their kneeling ability following different knee arthroplasty procedures, the AWT-K, being an objective kneeling test, seems well-suited to supplement patient-reported measures in future tibial nailing studies.

General study characteristics were also identified in this review. In-line with recent scoping reviews [58], no formal quality assessment into the level of evidence was conducted, yet still presented to give an indication into study quality. Most studies were level III evidence (49%), and retrospective studies were most prevalent (47%). There were few (5%) level I studies, echoing sentiments in the literature describing a lack of high quality RCTs in this area [59]. Though not explicitly recorded, information detailing the manner in which outcomes were administered, e.g., ROM and pain scores, was generally poor, as also highlighted in a review by Leliveld [17]. Surprisingly, almost a third (30%) of fractures in in vivo studies did not have a description of their nailing approach. The use of locking bolts, the fracture type/location and whether it is open/closed, the reaming technique, the post-operative weight bearing regime, and the mechanism of injury, are all important factors which may affect clinical and patient outcomes. Future studies should take care to report these items. In particular, the mechanism of injury and post-operative weight-bearing regime, which were not reported in most studies (55% and 60% of in vivo studies, respectfully), provide critical information into the energy of the injury and subsequent patient recovery. In terms of follow-up times, future prospective studies should aim for six weeks; and three-, six-, and 12-month time points (at a minimum) to adhere to those frequently reported.

Cadaveric outcomes assessing nail insertion location and damage to various structures is indicative of anatomical studies in this area, with a focus on identifying the safe nail entry zone, and detecting any iatrogenic structure damage. Intra-articular damage caused via SPN is considered one of the potential disadvantages of the approach, though some studies suggest that there is minimal risk [60]. These ex vivo studies provide important information often unobtainable from in vivo studies, hence their inclusion in this review.

There were a number of limitations to this study. Scoping reviews are general in nature, providing insight into current themes, but do not seek to critically appraise the results presented in the literature. Only English studies were included, most studies were low level of evidence (mostly level III (49%) or IV (30%)), and no adjustments were made to total fracture numbers to account for groups with multiple publications from the same cohort of patients. Further, only one researcher extracted the entirety of the relevant data, potentially biasing the results. However, the high level of overall agreement (94%) found from the data extraction check gives confidence in the results. The item with lowest agreement, the reported outcomes, was still moderate [61] (*k*-value = 0.43), even though its calculation was negatively skewed: if one assessor listed 9/10 outcomes, and the other 10/10, it would equate to complete disagreement for the outcomes extracted for that study.

Overall, the disparate nature of the outcomes in use necessitates a discussion to reach consensus on what outcomes should be reported, and what existing score(s) should be validated. Or, perhaps, highlights a compelling reason for the development of a score, or core outcome set [62], designed specifically for this cohort. The development or validation of a specific outcome measure would reduce the heterogeneity of outcomes reported, and provide a basis for future studies investigating different IM nailing techniques. A summary of recommendations from this scoping review is provided in Table 6.

**Table 6:** Recommendations for future studies investigating intramedullary nailing of tibial shaft fractures

Recommendations
Report the intramedullary nailing approach(es) used;
Specify the scale used for VAS and NRS outcomes, ideally a [0–10] or [0–100] scale;
Use notation to unambiguously and succinctly describe the specific outcome and its measurement scale, e.g., ${{VAS}_{AKP}}_{0}^{10}$;
Specify how the outcomes were administered, e.g., in-person or telephone;
Describe: the locking technique, the fracture type/location and if open/closed, the reaming technique, the post-operative weight bearing regime, and the mechanism of injury;
Prospective studies should aim for six weeks, and three-, six-, and 12-month follow-up time points;
Prospective studies should aim to include objective, performance-based outcome measures; and
Validation of a pain and/or function questionnaire specific to tibial nailing, which should include a kneeling component

*VAS* visual analogue scale, *NRS* numerical rating scale, *AKP* anterior knee pain

## Conclusion

This scoping review identified 179 studies investigating tibial shaft fractures treated with IM nailing. To our knowledge, this is the first study to comprehensively review and chart the clinical and patient outcomes used in this cohort. The lack of validated outcome measure designed specifically for this cohort has led to a wide range of outcomes in use. The most common patient and clinical outcomes were a binary (yes/no) assessment of knee pain (occurring in 29% of in vivo studies), and fracture union (52% of in vivo studies), respectively. Nail insertion location was the most reported outcome (66%) in ex vivo studies. A number of gaps in the literature were identified, in particular a lack of studies reporting objective, performance-based outcomes (only 7% of all in vivo studies). Future studies should take care to report key study information such as nail entry technique, which was not described in 30% of fractures in in vivo studies. The commonly reported outcomes identified in this scoping review should help reduce the heterogeneity of studies investigating IM nailing and ultimately help identify the optimal tibial nailing approach.

### Supplementary Information

Below is the link to the electronic supplementary material.

Supplementary file1 (PDF 96 KB)

Supplementary file2 (PDF 1258 KB)

Supplementary file3 (PDF 735 KB)
