# Publisher Correction: The absence of reporting standards and a lack of objective, performance-based outcomes following intramedullary nailing of tibial shaft fractures: findings from a scoping review into 179 articles

**DOI:** 10.1007/s00068-023-02380-z

**Published:** 2023-11-02

**Authors:** Simon Thwaites, John Abrahams, Dominic Thewlis, Mark Rickman

**Affiliations:** 1 Centre for Orthopaedic and Trauma Research, Adelaide Medical School, The University of Adelaide, Adelaide, South Australia Australia; 2 Department of Orthopaedics and Trauma, Royal Adelaide Hospital, Adelaide, South Australia Australia

**Publisher Correction: European Journal of Trauma and Emergency Surgery** 10.1007/s00068-023-02338-1

The original version of this article, published on 9 August 2023, unfortunately contained a mistake.

A production error converted a citation in Table 5 into a footnote, resulting in a number of in-text references to be out of sync with the appropriate citation number in the References section.

We apologize for the mistake.

The original article has been corrected.

