# Supplementary material for: The absence of reporting standards and a lack of objective, performance-based outcomes following intramedullary nailing of tibial shaft fractures: findings from a scoping review into 179 articles
Source: Eur J Trauma Emerg Surg. 2023 Aug 9;50(1):59–70. doi: 10.1007/s00068-023-02338-1 (PMC10924025; doi:10.1007/s00068-023-02338-1)
Supplement: Supplementary file 1 — Supplementary file1 (PDF 96 KB) [file 68_2023_2338_MOESM1_ESM.pdf]

Online Resource 1 – Protocol

Journal

European Journal of Trauma and Emergency Surgery

Title

The absence of reporting standards and a lack of objective, performance-based outcomes following intramedullary nailing of tibial shaft fractures: findings from a scoping review into 179 articles.

Authors

Simon Thwaites, John Abrahams, Dominic Thewlis, Mark Rickman

Corresponding author

Mr Simon Thwaites, M.Eng

Simon.thwaites@adelaide.edu.au

Centre for Orthopaedic & Trauma Research

Adelaide Health & Medical Sciences Building

4 North Terrace, ADELAIDE SA 5000

Centre for Orthopaedic & Trauma Research, Adelaide Medical School, The University of Adelaide, Adelaide, South Australia, Australia

ORCID: 0000-0001-9049-2165

Objectives

Summarise and map the outcomes used in literature reporting on tibial shaft fractures treated with intramedullary (IM) nailing. Provide details to comprehensively describe the nature of these publications.

Question:

What is known about the studies investigating tibial shaft fractures treated with IM nailing, and what outcomes are reported?

Table OR1.1: Inclusion and exclusion criteria

**Inclusion criteria**	**Exclusion criteria**
• Intramedullary nail fixation<br>• All suprapatellar and infrapatellar nailing approaches<br>• Tibial shaft fractures<br>• All populations<br>• All study designs (including cadaveric studies without presence of tibial shaft fracture)<br>• Original work<br>• English	• Review articles<br>• Non-tibial shaft fracture<br>• Open fractures only<br>• Other fracture fixation<br>• Intra-articular fractures<br>• Nail removal only presented<br>• Technical notes / technique description without a case series presented

PubMed search strategy:

Table OR1.2: Pubmed search strategy

Intramedullary nailing	tibia	Technique
“Fracture Fixation, Intramedullary”[mh]	“tibial fractures”[mh]	suprapatellar[tiab]
		OR infrapatellar[tiab]
OR “bone nails”[mh]	OR tibia shaft[tiab]	OR semiextended[tiab]
	OR tibial shaft[tiab]	OR retropatellar[tiab]
OR intramedullary nail*[tiab]	OR tibia*[tiab]	OR supra-patellar[tiab]
	OR fracture*[tiab]	OR infra-patellar[tiab]
		OR semi-extended[tiab]
		OR retro-patellar[tiab]
		OR medial parapatellar[tiab]
		OR lateral parapatellar[tiab]
		OR medial para-patellar[tiab]
		OR lateral para-patellar[tiab]
		OR transtendinous[tiab]

Embase search strategy:

Table OR1.3. Embase search strategy.

Intramedullary nailing	tibia	technique
intramedullary nail*/	tibial shaft/	suprapatellar intramedullary nailing/
OR fracture fixation*/	OR tibia shaft fracture/	OR infrapatellar intramedullary nailing/
OR bone nail/	OR tibia fracture/	OR semiextended intramedullary nailing/
OR intramedullary nail*.ti,ab	OR tibia* shaft.ti,ab	OR suprapatellar portal technique/
	OR tibia.ti,ab	OR infrapatellar tibial nail insertion/
	OR fracture.ti,ab	OR suprapatellar tibial nail insertion/
		OR suprapatellar.ti,ab
		OR infrapatellar.ti,ab
		OR semiextended.ti,ab
		OR retropatellar.ti,ab
		OR supra-patellar.ti,ab
		OR infra-patellar.ti,ab
		OR semi-extended.ti,ab
		OR retro-patellar.ti,ab

	OR medial parapatellar.ti,ab<br>OR lateral parapatellar.ti,ab<br>OR medial para-patellar.ti,ab<br>OR lateral para-patellar.ti,ab<br>OR transtendinous.ti,ab

Data Extraction

Use Covidence to screen papers and extract data. Use R to generate summary Tables and figures from exported covidence csv file. Search process:

Stage 1: Identify the research question

Stage 2: Identify relevant studies

Step 1: Initial limited search

Step 2: Identify key words and index terms

Step 3: Searching of references and citations

Stage 3: Study selection

Stage 4: Charting the data

Stage 5: Collating, summarising, and reporting the results

Data Extraction Template

General information

Year of publication

____

Author contact

____

Country in which the study conducted

1. United States

2. UK

3. Canada

4. Australia

5. Other

Notes

Level of Evidence

1. I

2. II

3. III

4. IV

5. V

6. Other

Study Characteristics

Nail focus

1. IPN

2. SPN

3. IPN vs SPN

4. NR

5. Other

Methods

Study design

1. Randomised controlled trial

2. Non-randomised experimental study

3. Prospective cohort study

4. Retrospective cohort study

5. Cross sectional study

6. Case control study

7. Case series

8. Case report

9. Cadaveric

10. Other

____

Approach 1

1. SPN midline

2. SPN medial

3. SPN lateral

4. SPN unspecified

5. Semi-extended lateral parapatellar

6. IPN transpatellar

7. IPN medial parapatellar

8. IPN lateral parapatellar

9. IPN unspecified

10. No description

11. Other

____

Approach 2

1. SPN midline
2. SPN medial
3. SPN lateral
4. SPN unspecified
5. Semi-extended lateral parapatellar
6. IPN transpatellar
7. IPN medial parapatellar
8. IPN lateral parapatellar
9. IPN unspecified
10. No description
11. NA
12. Other

Approach 3

1. SPN midline
2. SPN medial
3. SPN lateral
4. SPN unspecified
5. Semi-extended lateral parapatellar
6. IPN transpatellar
7. IPN medial parapatellar
8. IPN lateral parapatellar
9. IPN unspecified
10. No description

11. NA

12. Other

Locking description

1. Yes

2. No

3. NA

4. Other

Fracture description/location

1. Yes

2. No

3. NA

4. Other

Open/closed fracture description

1. Yes

2. No

3. NA

4. Other

Reamed description

1. Yes

2. No

3. NA

4. Other

Post-op weight bearing regime description

1. Yes

2. No

3. NA

4. Other

Nail removal

1. Yes

2. No

3. NA

4. Other

Mechanism of injury description

1. Yes

2. No

3. NA

4. Other

Participants

Total number of participants

Results

Outcomes used

1. Binary yes/no
2. Kujala
3. Lysholm
4. Oxford
5. IKCD
6. VAS
7. KOOS
8. WOMAC
9. QoL
10. SF-36
11. EQ5D
12. Surgery time
13. Blood loss
14. Radiation dose
15. Nail insertion location
16. Structure damage
17. Alignment
18. ROM
19. Other

Method of outcome

1. Phone
2. In person
3. Electronic
4. Email

5. Not-specified

6. Other

Participants

Total

Approach1

Approach2

Approach3

Other

Follow up times

time1 time2 time3 time4

Follow up time(s)
