# Supplementary material for: The absence of reporting standards and a lack of objective, performance-based outcomes following intramedullary nailing of tibial shaft fractures: findings from a scoping review into 179 articles
Source: Eur J Trauma Emerg Surg. 2023 Aug 9;50(1):59–70. doi: 10.1007/s00068-023-02338-1 (PMC10924025; doi:10.1007/s00068-023-02338-1)
Supplement: Supplementary file 2 — Supplementary file2 (PDF 1258 KB) [file 68_2023_2338_MOESM2_ESM.pdf]

Online Resource 2 – Other Results

Journal

European Journal of Trauma and Emergency Surgery

Title

The absence of reporting standards and a lack of objective, performance-based outcomes following intramedullary nailing of tibial shaft fractures: findings from a scoping review into 179 articles.

Authors

Simon Thwaites, John Abrahams, Dominic Thewlis, Mark Rickman

Corresponding author

Mr Simon Thwaites, M.Eng

Simon.thwaites@adelaide.edu.au

Centre for Orthopaedic & Trauma Research

Adelaide Health & Medical Sciences Building

4 North Terrace, ADELAIDE SA 5000

Centre for Orthopaedic & Trauma Research, Adelaide Medical School, The University of Adelaide, Adelaide, South Australia, Australia

ORCID: 0000-0001-9049-2165

Supplementary Table OR2.1. PubMed search strategy.

Intramedullary nailing	tibia	Technique
“Fracture Fixation, Intramedullary”[mh]	“tibial fractures”[mh]	suprapatellar[tiab]<br>OR infrapatellar[tiab]
OR “bone nails”[mh]	OR tibia* shaft[tiab]<br>OR tibia*[tiab]	OR semiextended[tiab]<br>OR retropatellar[tiab]
OR “intramedullary nail*”[tiab]	OR fracture*[tiab]	OR supra-patellar[tiab]<br>OR infra-patellar[tiab]<br>OR semi-extended[tiab]<br>OR retro-patellar[tiab]<br>OR medial parapatellar[tiab]<br>OR lateral parapatellar[tiab]<br>OR medial para-patellar[tiab]<br>OR lateral para-patellar[tiab]<br>OR transtendinous[tiab]

Supplementary Table OR2.2. Embase search strategy.

Intramedullary nailing	tibia	technique
intramedullary nail*/<br>OR fracture fixation*/<br>OR bone nail/<br><br>OR intramedullary nail*.ti,ab	tibial shaft/<br>OR tibia shaft fracture/<br>OR tibia fracture/<br><br>OR tibia* shaft.ti,ab<br>OR tibia.ti,ab<br>OR fracture.ti,ab	suprapatellar intramedullary nailing/<br>OR infrapatellar intramedullary nailing/<br>OR semiextended intramedullary nailing/<br>OR suprapatellar portal technique/<br>OR infrapatellar tibial nail insertion/<br>OR suprapatellar tibial nail insertion/<br><br>OR suprapatellar.ti,ab<br>OR infrapatellar.ti,ab<br>OR semiextended.ti,ab<br>OR retropatellar.ti,ab<br>OR supra-patellar.ti,ab<br>OR infra-patellar.ti,ab<br>OR semi-extended.ti,ab<br>OR retro-patellar.ti,ab<br>OR medial parapatellar.ti,ab<br>OR lateral parapatellar.ti,ab<br>OR medial para-patellar.ti,ab<br>OR lateral para-patellar.ti,ab<br>OR transtendinous.ti,ab

Supplementary Table OR2.3: Number of publications (n (%)) of grouped 'Other' countries from Table 2. 'Other' threshold < 4.

Country	n	(%)
Greece	3	(1.7)
Iran	3	(1.7)
The Netherlands	3	(1.7)
Brazil	2	(1.1)
Croatia	2	(1.1)
Japan	2	(1.1)
Nepal	2	(1.1)
Norway	2	(1.1)
Pakistan	2	(1.1)
Switzerland	2	(1.1)
Taiwan	2	(1.1)
Argentina	1	(0.6)
Denmark	1	(0.6)
France	1	(0.6)
Istanbul	1	(0.6)
Republic of Korea	1	(0.6)
Thailand	1	(0.6)

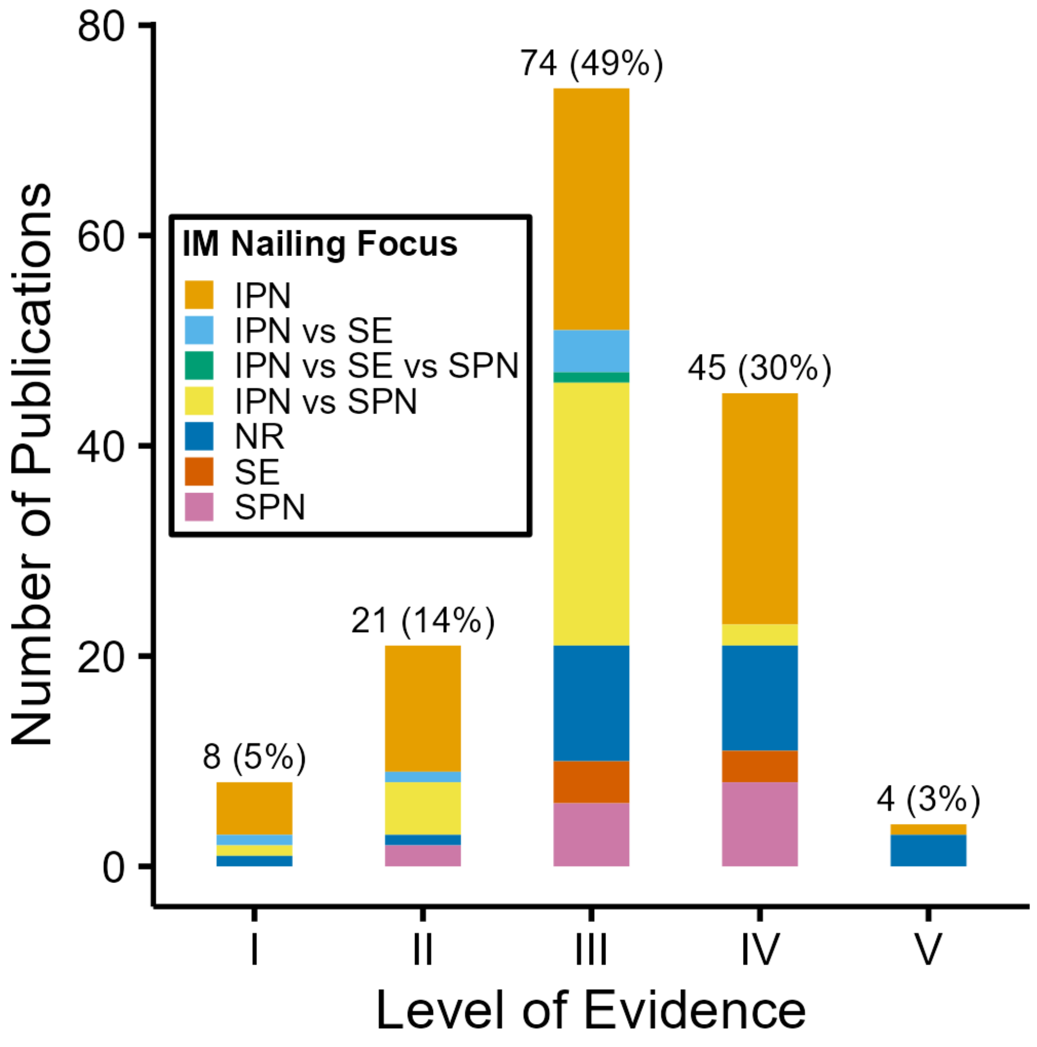

Supplementary Fig. OR2.1. Level of evidence grouped by intramedullary nailing focus. IM, intramedullary; IPN, infrapatellar nailing; SE, semi-extended; SPN, suprapatellar nailing; NR, not reported.

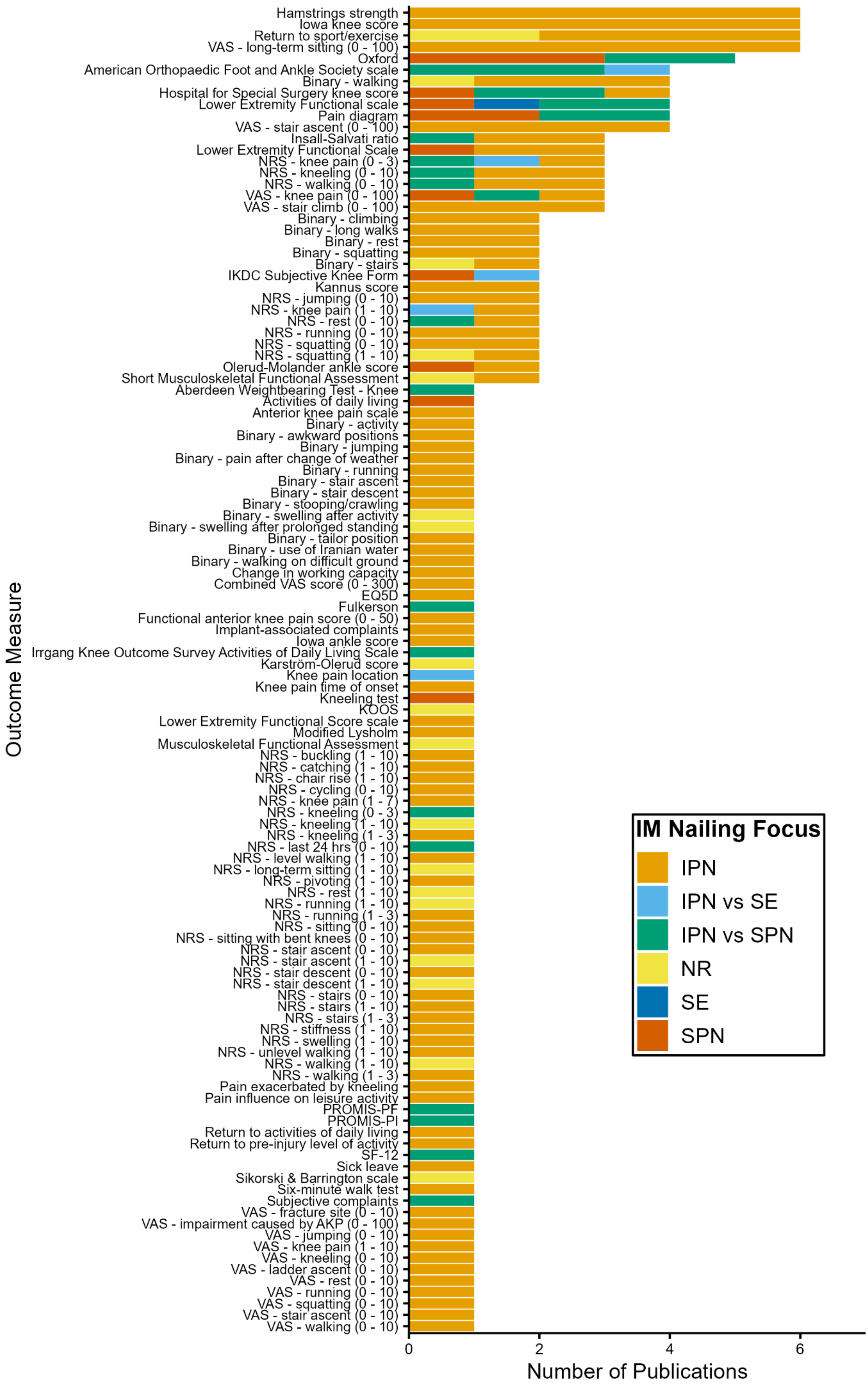
Hamstrings strength
Iowa knee score
Return to sport/exercise
VAS - long-term sitting (0 - 100)
Oxford
American Orthopaedic Foot and Ankle Society scale
Binary - walking
Hospital for Special Surgery knee score
Lower Extremity Functional scale
Pain diagram
VAS - stair ascent (0 - 100)
Insall-Salvati ratio
Lower Extremity Functional Scale
NRS - knee pain (0 - 3)
NRS - kneeling (0 - 10)
NRS - walking (0 - 10)
VAS - knee pain (0 - 100)
VAS - stair climb (0 - 100)
Binary - climbing
Binary - long walks
Binary - rest
Binary - squatting
Binary - stairs
IKDC Subjective Knee Form
Kannus score
NRS - jumping (0 - 10)
NRS - knee pain (1 - 10)
NRS - rest (0 - 10)
NRS - running (0 - 10)
NRS - squatting (0 - 10)
NRS - squatting (1 - 10)
Olerud-Molander ankle score
Short Musculoskeletal Functional Assessment
Aberdeen Weightbearing Test - Knee
Activities of daily living
Anterior knee pain scale
Binary - activity
Binary - awkward positions
Binary - jumping
Binary - pain after change of weather
Binary - running
Binary - stair ascent
Binary - stair descent
Binary - stooping/crawling
Binary - swelling after activity
Binary - swelling after prolonged standing
Binary - tailor position
Binary - use of Iranian water
Binary - walking on difficult ground
Change in working capacity
Combined VAS score (0 - 300)
EQ5D
Fulkerson
Functional anterior knee pain score (0 - 50)
Implant-associated complaints
Iowa ankle score
Irrgang Knee Outcome Survey Activities of Daily Living Scale
Karström-Olerud score
Knee pain location
Knee pain time of onset
Kneeling test
KOOS
Lower Extremity Functional Score scale
Modified Lysholm
Musculoskeletal Functional Assessment
NRS - buckling (1 - 10)
NRS - catching (1 - 10)
NRS - chair rise (1 - 10)
NRS - cycling (0 - 10)
NRS - knee pain (1 - 7)
NRS - kneeling (0 - 3)
NRS - kneeling (1 - 10)
NRS - kneeling (1 - 3)
NRS - last 24 hrs (0 - 10)
NRS - level walking (1 - 10)
NRS - long-term sitting (1 - 10)
NRS - pivoting (1 - 10)
NRS - rest (1 - 10)
NRS - running (1 - 10)
NRS - running (1 - 3)
NRS - sitting (0 - 10)
NRS - sitting with bent knees (0 - 10)
NRS - stair ascent (0 - 10)
NRS - stair ascent (1 - 10)
NRS - stair descent (0 - 10)
NRS - stair descent (1 - 10)
NRS - stairs (0 - 10)
NRS - stairs (1 - 10)
NRS - stairs (1 - 3)
NRS - stiffness (1 - 10)
NRS - swelling (1 - 10)
NRS - unlevel walking (1 - 10)
NRS - walking (1 - 10)
NRS - walking (1 - 3)
Pain exacerbated by kneeling
Pain influence on leisure activity
PROMIS-PF
PROMIS-PI
Return to activities of daily living
Return to pre-injury level of activity
SF-12
Sick leave
Sikorski & Barrington scale
Six-minute walk test
Subjective complaints
VAS - fracture site (0 - 10)
VAS - impairment caused by AKP (0 - 100)
VAS - jumping (0 - 10)
VAS - knee pain (1 - 10)
VAS - kneeling (0 - 10)
VAS - ladder ascent (0 - 10)
VAS - rest (0 - 10)
VAS - running (0 - 10)
VAS - squatting (0 - 10)
VAS - stair ascent (0 - 10)
VAS - walking (0 - 10)
Outcome Measure
0
2
4
6
Number of Publications
IM Nailing Focus
IPN
IPN vs SE
IPN vs SPN
NR
SE
SPN

Supplementary Fig. OR2.2: ‘Other’ patient outcomes for *in vivo* studies grouped by intramedullary nailing focus from Fig. 3 of main manuscript. 'Other' threshold < 7. IM, intramedullary; IPN, infrapatellar nailing; SE, semi-extended; SPN, suprapatellar nailing; NR, not reported; VAS, visual analogue scale; NRS, numerical rating scale; IKDC, International Knee Documentation Committee; KOOS, Knee Injury and Osteoarthritis Outcome Score; CT, computed tomography; PROMIS, patient-reported outcomes measurement information system; PF, physical function; PI, pain interference; SF-12, 12-item short form health survey; AKP, anterior knee pain.

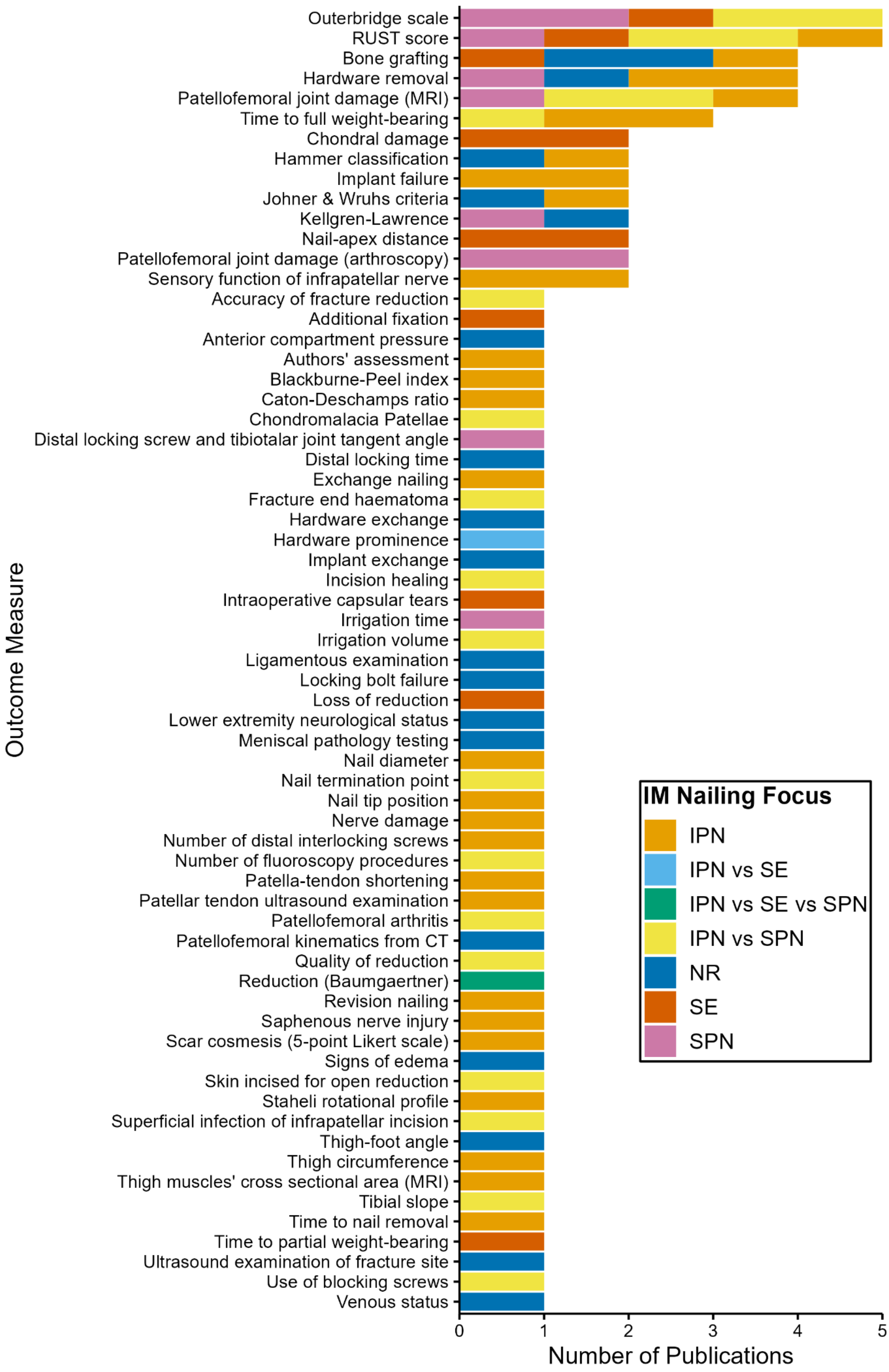

Outerbridge scale
RUST score
Bone grafting
Hardware removal
Patellofemoral joint damage (MRI)
Time to full weight-bearing
Chondral damage
Hammer classification
Implant failure
Johner & Wruhs criteria
Kellgren-Lawrence
Nail-apex distance
Patellofemoral joint damage (arthroscopy)
Sensory function of infrapatellar nerve
Accuracy of fracture reduction
Additional fixation
Anterior compartment pressure
Authors' assessment
Blackburne-Peel index
Caton-Deschamps ratio
Chondromalacia Patellae
Distal locking screw and tibiotalar joint tangent angle
Distal locking time
Exchange nailing
Fracture end haematoma
Hardware exchange
Hardware prominence
Implant exchange
Incision healing
Intraoperative capsular tears
Irrigation time
Irrigation volume
Ligamentous examination
Locking bolt failure
Loss of reduction
Lower extremity neurological status
Meniscal pathology testing
Nail diameter
Nail termination point
Nail tip position
Nerve damage
Number of distal interlocking screws
Number of fluoroscopy procedures
Patella-tendon shortening
Patellar tendon ultrasound examination
Patellofemoral arthritis
Patellofemoral kinematics from CT
Quality of reduction
Reduction (Baumgaertner)
Revision nailing
Saphenous nerve injury
Scar cosmesis (5-point Likert scale)
Signs of edema
Skin incised for open reduction
Staheli rotational profile
Superficial infection of infrapatellar incision
Thigh-foot angle
Thigh circumference
Thigh muscles' cross sectional area (MRI)
Tibial slope
Time to nail removal
Time to partial weight-bearing
Ultrasound examination of fracture site
Use of blocking screws
Venous status
Outcome Measure
0
1
2
3
4
5
Number of Publications
IM Nailing Focus
IPN
IPN vs SE
IPN vs SE vs SPN
IPN vs SPN
NR
SE
SPN

Supplementary Fig. OR2.3: ‘Other’ clinical outcomes for *in vivo* studies grouped by intramedullary nailing focus from Fig. 4 of main manuscript. 'Other' threshold < 6. IM, intramedullary; IPN, infrapatellar nailing; SE, semi-extended; SPN, suprapatellar nailing; NR, not reported; RUST, radiographic union score for tibia; MRI, magnetic resonance imaging.

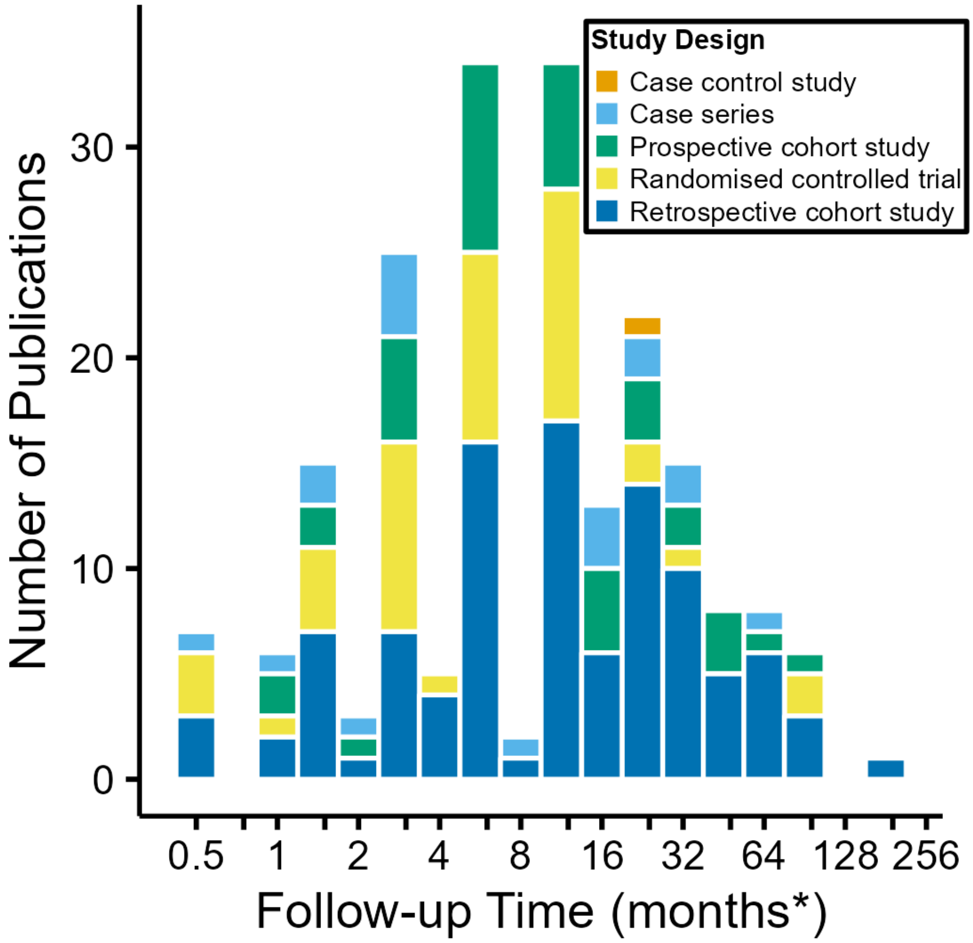

Supplementary Fig. OR2.4: Follow-up times (in months) for intramedullary nailing studies grouped by study design. *log2 scale.

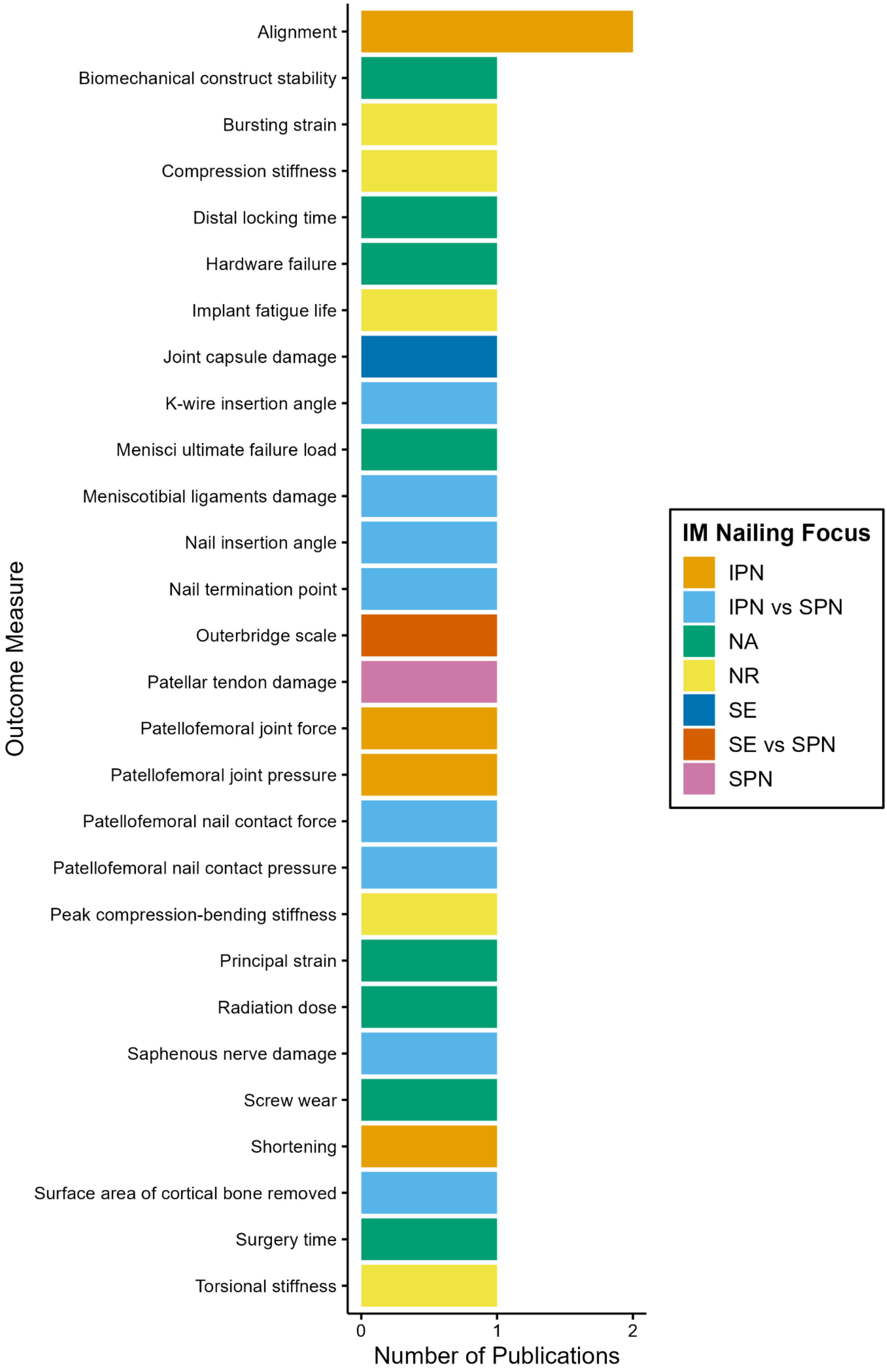
Outcome Measure
Alignment
Biomechanical construct stability
Bursting strain
Compression stiffness
Distal locking time
Hardware failure
Implant fatigue life
Joint capsule damage
K-wire insertion angle
Menisci ultimate failure load
Meniscotibial ligaments damage
Nail insertion angle
Nail termination point
Outerbridge scale
Patellar tendon damage
Patellofemoral joint force
Patellofemoral joint pressure
Patellofemoral nail contact force
Patellofemoral nail contact pressure
Peak compression-bending stiffness
Principal strain
Radiation dose
Saphenous nerve damage
Screw wear
Shortening
Surface area of cortical bone removed
Surgery time
Torsional stiffness
0
1
2
Number of Publications
IM Nailing Focus
IPN
IPN vs SPN
NA
NR
SE
SE vs SPN
SPN

Supplementary Fig. OR2.5: ‘Other’ outcomes for cadaveric studies grouped by intramedullary nailing focus from Fig. 6 of main manuscript. 'Other' threshold < 3. IM, intramedullary; IPN, infrapatellar nailing; SE, semi-extended; SPN, suprapatellar nailing; NA, not applicable; NR, not reported.
