# Supplementary material for: The absence of reporting standards and a lack of objective, performance-based outcomes following intramedullary nailing of tibial shaft fractures: findings from a scoping review into 179 articles
Source: Eur J Trauma Emerg Surg. 2023 Aug 9;50(1):59–70. doi: 10.1007/s00068-023-02338-1 (PMC10924025; doi:10.1007/s00068-023-02338-1)
Supplement: Supplementary file 3 — Supplementary file3 (PDF 735 KB) [file 68_2023_2338_MOESM3_ESM.pdf]

Online Resource 3 – Included studies and extracted data

Journal

European Journal of Trauma and Emergency Surgery

Title

The absence of reporting standards and a lack of objective, performance-based outcomes following intramedullary nailing of tibial shaft fractures: findings from a scoping review into 179 articles.

Authors

Simon Thwaites, John Abrahams, Dominic Thewlis, Mark Rickman

Corresponding author

Mr Simon Thwaites, M.Eng

Simon.thwaites@adelaide.edu.au

Centre for Orthopaedic & Trauma Research

Adelaide Health & Medical Sciences Building

4 North Terrace, ADELAIDE SA 5000

Centre for Orthopaedic & Trauma Research, Adelaide Medical School, The University of Adelaide, Adelaide, South Australia, Australia

ORCID: 0000-0001-9049-2165

TABLE OR3.1. Included studies and extracted data. Lock., locking; Frac. Desc./ Loc., fracture description/location; O/C, open/closed, Ream., reaming; PO WB, poste-operative weight-bearing; MOI, mechanism of injury; Nail rem., nail removal; n_{A1}, Approach 1 numbers; n_{A2}, Approach 2 numbers; n_{A3}, Approach 3 numbers; n_{Tot}, total number of fractures (*in vivo* studies) or tibias (*ex vivo* studies) included; LOE, level of evidence; SPN, suprapatellar nailing; IPN, infrapatellar nailing; SE, semi-extended; ROM, range of motion; EA, extra-articular; IA, intra-articular; VAS, visual analogue scale; ND, no description; SF-36, 36-Item Short Form Health Survey; MRI, magnetic resonance imaging; RUST, Radiographic Union Scale in Tibial Fractures; NRS, numerical rating scale; PROMIS-PI, patient-reported outcomes measurement information system-pain interference; PROMIS-PF, patient-reported outcomes measurement information system-physical function; IKDC, International Knee Documentation Committee; NR, not reported; KOOS, The Knee Injury and Osteoarthritis Outcome Score.

Study	Country	Study design	Outcomes used	Nail focus	Approach 1	Approach 2	Approach 3	n_{A1}	n_{A2}	n_{A3}	n_{Tot}	Lock.	Frac. Desc./ Loc.	O/C	Ream.	PO WB	MOI	Nail Rem.	LOE	Follow up time(s) (mths)
Sahni 2023[1]	India	Prospective cohort study	Surgery time, Blood loss, Radiation dose, Union, Lower Extremity Functional scale, VAS - knee pain (ND)	IPN vs SPN	IPN transpatellar	SPN midline	-	20	20	-	40	No	Yes	Yes	No	Yes	No	No	II	1.5, 3, 6
Gupta 2023[2]	India	Retrospective cohort study	Lysholm, Surgery time, Blood loss, Alignment, American Orthopaedic Foot and Ankle Society scale, VAS - knee pain (ND), Union	IPN vs SPN	IPN transpatellar	SPN unspecified	-	36	27	-	63	Yes	No	No	No	No	No	No	III	12
Chahal 2022[3]	India	Prospective cohort study	Lysholm, Surgery time, Union, Lower Extremity Functional scale, VAS - knee pain (ND), Time to full weight-bearing, Complications	IPN vs SPN	IPN transpatellar	SPN unspecified	-	15	15	-	30	No	Yes	Yes	No	Yes	Yes	No	II	1, 2, 3, 6
Lu 2022[4]	China	Randomised controlled trial	Surgery time, Blood loss, Radiation dose, Alignment, Hospital stay, Complications, Union, American Orthopaedic Foot and Ankle Society scale, Infection	IPN vs SE	IPN transpatellar	Semi-extended lateral paratendinous (EA)	-	45	43	-	88	Yes	Yes	Yes	Yes	Yes	No	No	II	3, 6, 12

Leliveld 2022[5]	The Netherlands	Randomised controlled trial	EQ5D, Surgery time, Alignment, ROM, Sensory function of infrapatellar nerve, NRS - kneeling (0 - 10), NRS - walking (0 - 10), NRS - running (0 - 10), NRS - jumping (0 - 10), NRS - stair ascent (0 - 10), NRS - stair descent (0 - 10), NRS - squatting (0 - 10), NRS - sitting (0 - 10), NRS - cycling (0 - 10), Lower Extremity Functional Score scale, Nail prominence, Short Musculoskeletal Functional Assessment, Scar cosmesis (5-point Likert scale), Return to work, Return to activities of daily living, Complications, Infection, Hospital stay	IPN	IPN transpatellar (transverse incision)	IPN transpatellar (longitudinal incision)	-	68	68	-	136	No	Yes	Yes	Yes	No	Yes	Yes	I	0.5, 1.5, 3, 6, 12
Basile 2022[6]	Italy	Case series	Binary yes/no, Complications, Union	SPN	SPN midline	-	-	2	-	-	2	No	Yes	No	Yes	Yes	No	No	IV	-
He 2022[7]	China	Retrospective cohort study	Alignment, Distal locking screw and tibiotalar joint tangent angle	SPN	SPN unspecified	-	-	100	-	-	100	Yes	Yes	Yes	No	No	Yes	No	III	-
Sathy 2022[8]	United States	Retrospective cohort study	Alignment, Number of distal interlocking screws	IPN	IPN grouped transpatellar and medial/lateral parapatellar	-	-	81	-	-	81	Yes	Yes	Yes	Yes	No	Yes	No	III	-
Yasuda 2022[9]	Japan	Retrospective cohort study	Surgery time, Blood loss, Nail insertion location, Alignment, ROM, Union, Infection, Chondral damage, Outerbridge scale, Intraoperative	SE	Semi-extended medial parapatellar (EA)	Semi-extended medial parapatellar (IA)	-	29	25	-	54	No	Yes	Yes	Yes	Yes	Yes	No	III	12

			capsular tears, Loss of reduction																	
Approach 2022[10]	India	Prospective cohort study	Binary yes/no, Lower Extremity Functional scale, Hospital stay, Union, Complications	SPN	SPN midline	-	-	20	-	-	20	Yes	No	Yes	Yes	No	No	No	III	1, 6, 12
Gao 2022[11]	China	Retrospective cohort study	Lysholm, VAS - knee pain (0 - 10), Surgery time, Union, Complications, Incision healing, American Orthopaedic Foot and Ankle Society scale, Alignment, Number of fluoroscopy procedures, Use of blocking screws, Skin incised for open reduction, Fracture end haematoma, Superficial infection of infrapatellar incision, Hardware failure	IPN vs SPN	SPN midline	IPN medial parapatellar	-	69	63	-	132	Yes	Yes	Yes	Yes	Yes	Yes	No	III	6, 12
Llano 2022[12]	Argentina	Retrospective cohort study	Lysholm, Surgery time, Radiation dose, Alignment, VAS - knee pain (ND)	IPN vs SPN	SPN unspecified	IPN unspecified	-	44	36	-	80	No	Yes	No	No	No	No	No	III	1, 12
Patel 2022[13]	United States	Cadaveric	Nail insertion location, Joint capsule damage	SE	Semi-extended lateral parapatellar (EA)	-	-	6	-	-	6	NA	NA	NA	Yes	NA	NA	No	NA	-
Umur 2022[14]	Turkey	Retrospective cohort study	Binary yes/no, Lysholm, SF-36, Surgery time, ROM, Patellofemoral joint damage (MRI), Union, VAS - knee pain (ND), RUST score, Outerbridge scale	IPN vs SPN	SPN midline	IPN medial parapatellar	-	29	32	-	61	Yes	Yes	Yes	No	Yes	No	No	III	6
Al-Azzawi 2021[15]	UK	Case series	Lysholm, Surgery time, Radiation dose, Nail insertion location	IPN vs SPN	SPN unspecified	IPN unspecified	-	22	20	-	42	No	Yes	Yes	No	No	Yes	No	IV	1.5, 3,6
Baker 2021[16]	United States	Retrospective cohort study	Alignment, Union, Reduction (Baumgaertner)	IPN vs SE vs SPN	IPN unspecified	Semi-extended lateral parapatellar (EA)	SPN unspecified	17	19	21	57	Yes	Yes	Yes	No	No	Yes	No	III	1.5
Daley-Lindo 2021[17]	United States	Retrospective cohort study	Tegner	SPN	SPN midline	-	-	35	-	-	35	Yes	Yes	Yes	Yes	Yes	No	Yes	III	60

Fontalis 2021[18]	UK	Retrospective cohort study	Binary yes/no, Kujala, Oxford, NRS - knee pain (0 - 3), NRS - kneeling (0 - 3), Binary - kneeling	IPN vs SPN	SPN midline	IPN transpatellar	-	61	41	-	102	No	Yes	No	No	No	No	No	III	6
Graulich 2021[19]	Germany	Retrospective cohort study	Kujala, Lysholm, Insall-Salvati ratio, Nail tip position	IPN	IPN transpatellar	-	-	78	-	-	78	Yes	Yes	No	Yes	No	No	No	III	1.5, 3, 6, 12
Hague 2021[20]	UK	Retrospective cohort study	Alignment	IPN vs SPN	SPN unspecified	IPN unspecified	-	74	51	-	125	No	No	Yes	No	No	No	No	III	-
Khan 2021[21]	UK	Retrospective cohort study	VAS - knee pain (ND)	IPN	IPN transpatellar	IPN medial parapatellar	-	30	30	-	60	No	No	Yes	No	No	No	No	IV	3
Lu 2021[22]	China	Retrospective cohort study	Binary yes/no, Lysholm, VAS - knee pain (0 - 10), SF-36, Surgery time, Radiation dose, Alignment, Union, Hospital stay, Infection	IPN vs SE	Semi-extended lateral paratendinous (EA)	IPN transpatellar	-	40	40	-	80	Yes	Yes	Yes	Yes	Yes	Yes	No	III	1, 2, 3, 6, 12
Metcalf 2021[23]	United States	Retrospective cohort study	Binary yes/no, Union, Infection, PROMIS-PI, PROMIS-PF	IPN vs SPN	SPN unspecified	IPN unspecified	-	107	101	-	208	No	Yes	Yes	No	No	Yes	No	III	3, 6, 12
Shi 2021[24]	United States	Cadaveric	Saphenous nerve damage	IPN vs SPN	SPN midline	IPN medial parapatellar	-	6	8	-	14	Yes	NA	NA	Yes	NA	NA	No	NA	-
Zhao 2021[25]	China	Retrospective cohort study	Binary yes/no, Surgery time, Blood loss, Radiation dose, Alignment, RUST score, Lower Extremity Functional Scale, Infection, Complications, Rotational deformity	IPN	IPN unspecified	-	-	112	-	-	112	Yes	Yes	Yes	Yes	Yes	Yes	No	III	12
Zhu 2021[26]	China	Retrospective cohort study	Lysholm, SF-36, ROM, Hospital for Special Surgery knee score, VAS - knee pain (ND)	IPN vs SPN	SPN midline	IPN transpatellar	-	55	55	-	110	Yes	No	No	No	Yes	Yes	No	III	6
Xu 2020[27]	China	Case series	Surgery time, Radiation dose, Alignment, Union	IPN	IPN unspecified	-	-	36	-	-	36	Yes	Yes	Yes	Yes	No	No	No	IV	12
Singh 2020[28]	UK	Case series	Kujala, Oxford, VAS - knee pain (0 - 100), Union	SPN	SPN lateral	-		22	-	-	22	Yes	No	Yes	Yes	No	No	No	IV	60
Lu 2020[29]	China	Retrospective cohort study	Lysholm, Surgery time, Blood loss, Union, Complications, American Orthopaedic Foot and Ankle Society scale, VAS - knee pain (ND)	IPN vs SPN	SPN midline	IPN transpatellar	-	27	36	-	63	Yes	Yes	No	No	No	No	No	III	12

Lu 2020[30]	China	Retrospective cohort study	Binary yes/no, Lysholm, Surgery time, Blood loss, Radiation dose, Alignment, Complications, Irrigation volume, Pain diagram	IPN vs SPN	SPN unspecified	IPN medial parapatellar	-	38	43	-	81	Yes	Yes	Yes	No	No	Yes	No	III	12
Kulkarni 2020[31]	India	Retrospective cohort study	Binary yes/no, Alignment, Union, Lower Extremity Functional Scale, Shortening, Rotational deformity	SPN	SPN midline	-	-	43	-	-	43	Yes	Yes	Yes	Yes	No	No	No	IV	1.5, 3, 4.5, 6, 12
Cinats 2020[32]	Canada	Retrospective cohort study	Surgery time, Nail insertion location, Alignment, Union	IPN vs SPN	IPN transpatellar, IPN medial parapatellar	SPN midline	-	22	23	-	45	Yes	No	No	Yes	Yes	No	No	III	1.5
Allen 2020[33]	United States	Retrospective cohort study	Surgery time, Radiation dose, Alignment	IPN vs SPN	SPN unspecified	IPN unspecified	-	177	164	-	341	Yes	Yes	Yes	No	No	No	No	III	-
Ozcan 2020[34]	Turkey	Prospective cohort study	Binary yes/no, Kujala, Lysholm, Surgery time, Nail insertion location, Hospital stay	IPN vs SPN	SPN midline	IPN transpatellar	IPN medial parapatellar	21	21	16	58	Yes	No	No	Yes	No	No	No	III	16, 23, 45
Maslow 2020[35]	United States	Retrospective cohort study	Nail insertion location, ROM	IPN vs SPN	SPN unspecified	IPN unspecified	-	50	50	-	100	No	Yes	Yes	No	No	No	No	III	-
Lu 2020[36]	China	Randomised controlled trial	Lysholm, VAS - knee pain (0 - 10), Alignment, ROM, Union, Patellofemoral joint damage (MRI), Pain diagram, Rotational deformity	SPN	SPN unspecified	SPN Chinese aircraft-shaped sleeve	-	34	33	-	67	Yes	Yes	Yes	No	No	Yes	Yes	II	12
Hessmann 2020[37]	Germany	Retrospective cohort study	Surgery time, Alignment, ROM, Union	SPN	SPN unspecified	-	-	61	-	-	61	Yes	Yes	Yes	No	No	Yes	No	III	-
Valsamis 2019[38]	UK	Prospective cohort study	Radiation dose	IPN vs SPN	IPN transpatellar	SPN midline	-	43	40	-	83	No	Yes	No	No	No	No	No	III	-
Stella 2019[39]	Italy	Prospective cohort study	Binary yes/no, Lysholm, Alignment, ROM, Nail-apex distance, Union, RUST score, Quadriceps strength, Complications, VAS - knee pain (ND)	SE	Semi-extended lateral parapatellar (EA)	-	-	70	-	-	70	Yes	Yes	Yes	Yes	Yes	No	No	III	41.2
Serbest 2019[40]	Turkey	Prospective cohort study	Lysholm, SF-36, Radiation dose, Nail insertion location, Alignment, ROM, Union, Outerbridge	SPN	SPN unspecified	-	-	21	-	-	21	No	Yes	Yes	No	Yes	No	No	IV	3,6,12

			scale, VAS - knee pain (ND), Patellofemoral joint damage (arthroscopy), Rotational deformity																	
Rothberg 2019[41]	United States	Randomised controlled trial	Alignment, Union, Hardware prominence, IKDC Subjective Knee Form	IPN vs SE	Semi-extended lateral parapatellar (EA)	IPN unspecified	-	24	23	-	47	Yes	Yes	Yes	Yes	No	Yes	Yes	I	1.5, 3, 6, 12
Özbek 2019[42]	Turkey	Retrospective cohort study	Binary yes/no, Quadriceps strength, Hamstrings strength, Staheli rotational profile, Tegner	IPN	IPN transpatellar	-	-	40	-	-	40	No	Yes	Yes	No	No	Yes	No	III	22.5
Nicolescu 2019[43]	United States	Retrospective cohort study	Alignment	IPN vs SPN	SPN unspecified	IPN transpatellar	-	42	35	-	77	Yes	Yes	Yes	No	No	No	No	III	-
MacDonald 2019[44]	UK	Randomised controlled trial	Lysholm, Surgery time, Radiation dose, Aberdeen Weightbearing Test - Knee, Irrgang Knee Outcome Survey Activities of Daily Living Scale, Hospital stay, Complications, VAS - knee pain (0 - 100), Fulkerson	IPN vs SPN	SPN unspecified	IPN medial parapatellar	-	53	42	-	95	No	No	Yes	No	No	Yes	No	I	4, 6, 12
Ladurner 2019[45]	Switzerland	Retrospective cohort study	Surgery time, Radiation dose, Hospital stay, Complications	IPN vs SE	IPN transpatellar	IPN medial parapatellar	Semi-extended lateral parapatellar (EA)	29	18	26	73	Yes	Yes	Yes	Yes	No	Yes	No	III	-
Isaac 2019[46]	United States	Retrospective cohort study	NRS - kneeling (0 - 10), NRS - rest (0 - 10), NRS - walking (0 - 10), NRS - last 24 hrs (0 - 10)	IPN vs SPN	IPN medial parapatellar	IPN transpatellar	SPN midline	171	-	91	262	No	No	Yes	No	No	No	No	III	43.2, 50.4
Cui 2019[47]	China	Retrospective cohort study	Surgery time, Blood loss, Hospital for Special Surgery knee score	IPN vs SPN	IPN medial parapatellar	SPN midline	-	26	24	-	50	No	No	Yes	Yes	No	No	No	III	23.08, 23.92
Çiçekli 2019[48]	Turkey	Retrospective cohort study	Kujala, Lysholm, Surgery time, Radiation dose, Nail insertion location, Alignment, Insall-Salvati ratio, Patellofemoral arthritis, Tibial slope, Union	IPN vs SPN	SPN midline	IPN unspecified	-	33	41	--	74	No	Yes	Yes	No	No	No	No	III	29.21, 30.2
Çiçekli 2019[49]	Turkey	Retrospective cohort study	Lysholm, Alignment,	SPN	SPN midline	-	-	58	-	-	58	Yes	Yes	Yes	No	Yes	No	No	III	19.83

			Kellgren-Lawrence, Union, VAS - knee pain (ND)																	
Leliveld 2019[50]	The Netherlands	Randomised controlled trial	NRS - kneeling (0 - 10), NRS - squatting (0 - 10), NRS - sitting with bent knees (0 - 10), NRS - jumping (0 - 10), NRS - stairs (0 - 10), NRS - running (0 - 10), NRS - rest (0 - 10), NRS - walking (0 - 10)	IPN	IPN medial parapatellar	IPN transpatellar	-	17	17	-	34	No	Yes	Yes	No	No	No	Yes	II	86
Anderson 2019[51]	UK	Retrospective cohort study	Nail insertion location	IPN vs SPN	IPN unspecified	SPN unspecified	-	105	95	-	200	No	No	No	No	No	No	No	III	-
Williamson 2018[52]	UK	Retrospective cohort study	Radiation dose	IPN vs SPN	IPN transpatellar	SPN midline	-	37	53	-	90	No	No	No	No	No	No	No	III	-
Tajima 2018[53]	Japan	Case series	Surgery time	SPN	SPN midline	-	-	44	-	-	44	No	No	No	Yes	No	No	No	IV	-
Schumaier 2018[54]	United States	Prospective cohort study, Cadaveric	Nail insertion location, Nail termination point	IPN vs SPN	SPN unspecified	IPN transpatellar	IPN medial parapatellar	4	4	6	14	No	Yes	No	No	No	No	No	II	6
Lu 2018[55]	China	Randomised controlled trial	Surgery time, Outerbridge scale, Irrigation time	SPN	SPN midline	-	-	30	30	-	60	Yes	No	No	Yes	No	No	No	II	-
Franke 2018[56]	Germany	Cadaveric	Nail insertion location, K-wire insertion angle, Nail insertion angle	IPN vs SPN	SPN midline	IPN transpatellar	-	19	17	-	36	No	NA	NA	Yes	NA	NA	No	NA	-
Cazzato 2018[57]	Italy	Case series	Kujala, Lysholm, Oxford, SF-36, Alignment, ROM, Infection, Hardware removal, IKDC Subjective Knee Form	SPN	SPN unspecified	-	-	25	-	-	25	Yes	Yes	Yes	No	No	No	No	IV	28.76
Triantafillou 2017[58]	United States	Retrospective cohort study	Alignment	IPN vs SPN	IPN medial parapatellar	SPN unspecified	-	64	21	-	85	Yes	Yes	Yes	Yes	No	No	No	IV	-
Turkmen 2017[59]	Turkey	Retrospective cohort study	Binary yes/no, Lysholm, Nail insertion location, Alignment, ROM, Hospital for Special Surgery knee score, Thigh circumference, Union, Nail prominence, Insall-Salvati ratio, Caton-Deschamps ratio	IPN	IPN transpatellar	-	-	33	-	-	33	Yes	Yes	Yes	Yes	Yes	Yes	Yes	III	79.2
Mehta 2017[60]	UK	Retrospective cohort study	Alignment, Union, RUST score	IPN vs SPN	SPN unspecified	IPN unspecified	-	20	20	-	40	Yes	Yes	No	No	No	No	No	III	6

Soraganvi 2016[61]	India	Retrospective cohort study	Binary yes/no, Nail insertion location, Nail prominence	IPN	IPN transpatellar	-	-	103	-	-	103	Yes	No	No	No	No	No	No	IV	24, 30
De Giacomo 2016[62]	United States	Retrospective cohort study	Alignment, Union, Complications	NR	No description	-	-	122	-	-	122	Yes	Yes	Yes	Yes	Yes	Yes	Yes	IV	
Zamora 2016[63]	United States	Cadaveric	Nail insertion location, Outerbridge scale, Menisci damage (inspection post-dissection), Anterior cruciate ligament damage (inspection post-dissection), Intermeniscal ligament damage (inspection post-dissection), Articular surface damage (inspection post-dissection)	SE vs SPN	Semi-extended medial parapatellar (EA)	Semi-extended lateral parapatellar (EA)	SPN midline	5	5	10	20	NA	NA	NA	Yes	NA	NA	Yes	NA	-
Sun 2016[64]	China	Randomised controlled trial	Binary yes/no, Lysholm, Surgery time, Blood loss, Radiation dose, ROM, Hospital stay, Complications, Union, VAS - knee pain (ND), Patellofemoral joint damage (MRI)	IPN vs SPN	SPN midline	IPN medial parapatellar	-	75	74	-	149	Yes	Yes	Yes	Yes	No	No	Yes	II	3 ,6, 12, 24
Obremskey 2016[65]	United States	Prospective cohort study	NRS - knee pain (1 - 7), NRS - kneeling (1 - 3), NRS - stairs (1 - 3), NRS - walking (1 - 3), NRS - running (1 - 3)	IPN	IPN medial parapatellar	IPN transpatellar	-	337	100	-	437	Yes	Yes	Yes	No	No	No	No	II	3, 6, 12
Fu 2016[66]	China	Retrospective cohort study	Surgery time, Blood loss, Radiation dose, ROM, Union, Hospital for Special Surgery knee score, Olerud-Molander ankle score, Hospital stay	SPN	SPN midline	-	-	23	-	-	23	Yes	Yes	Yes	Yes	Yes	No	No	III	15.5
Chan 2016[67]	United States	Randomised controlled trial	Lysholm, VAS - knee pain (0 - 10), SF-36, Alignment, ROM, Outerbridge scale, Pain diagram, Subjective complaints, Union,	IPN vs SPN	IPN medial parapatellar	SPN midline	-	14	11	-	25	Yes	Yes	Yes	Yes	No	No	Yes	II	1.5, 3, 6, 12

			Chondromalacia Patellae																	
Bakhsh 2016[68]	United States	Retrospective cohort study	Lysholm, Knee pain location, NRS - knee pain (1 - 10), Complications	IPN vs SE	Semi-extended lateral parapatellar (EA)	IPN medial parapatellar	IPN transpatellar	34	34	34	102	No	No	Yes	No	No	Yes	No	III	31, 61, 65
Avilucea 2016[69]	United States	Retrospective cohort study	Alignment	IPN vs SPN	SPN unspecified	IPN grouped transpatellar and medial/lateral parapatellar	-	132	134	-	266	Yes	Yes	Yes	No	No	No	No	III	-
Aksahin 2016[70]	Turkey	Case series	Patellofemoral joint damage (MRI), Thigh muscles' cross sectional area (MRI)	IPN	IPN unspecified	-	-	27	-	-	27	Yes	No	No	No	No	No	No	IV	28.3
Ahmad 2016[71]	Pakistan	Randomised controlled trial	Binary yes/no, VAS	IPN	IPN transpatellar	IPN medial parapatellar	-	30	30	-	60	No	No	Yes	No	No	Yes	No	II	3
LaPrade 2015[72]	United States	Cadaveric	Menisci damage, Menisci ultimate failure load	NA	NA	-	-	12	-	-	12	No	NA	NA	Yes	NA	NA	No	NA	-
Kruppa 2015[73]	United States	Retrospective cohort study	Binary yes/no, Alignment, ROM, Union, Implant-associated complaints, Complications, Infection, Shortening, Return to work	IPN	IPN transpatellar	-	-	105	-	-	105	Yes	Yes	Yes	Yes	Yes	Yes	No	IV	0.5, 1.5, 4.5, 6, 12
Courtney 2015[74]	United States	Retrospective cohort study	Binary yes/no, Oxford, Surgery time, Radiation dose, Union, Quality of reduction, Rotational deformity	IPN vs SPN	SPN unspecified	IPN unspecified	-	21	24	-	45	No	Yes	Yes	No	No	Yes	No	III	12, 25
Tonk 2014[75]	India	Randomised controlled trial	Binary yes/no, Lysholm, Union, VAS - knee pain (0 - 100)	IPN	IPN transpatellar	IPN medial parapatellar	-	28	28	-	56	Yes	Yes	No	Yes	No	Yes	No	V	0.5, 1.5, 3, 6
Say 2014[76]	Turkey	Retrospective cohort study	Rotational deformity, Thigh-foot angle	NR	No description	-	-	26	-	-	26	No	Yes	Yes	Yes	Yes	No	No	IV	-
Larsen 2014[77]	Denmark	Retrospective cohort study	KOOS, Complications, Reoperation, Compartment syndrome	NR	No description	-	-	223	-	-	223	Yes	Yes	Yes	Yes	No	No	Yes	III	94.8
Tahririan 2014[78]	Iran	Retrospective cohort study	Binary yes/no, Nail insertion location, Binary - walking, Binary - running, Binary - squatting, Binary - kneeling	IPN	IPN transpatellar	IPN medial parapatellar	-	35	60	-	95	Yes	No	Yes	No	No	No	No	IV	-
Sanders 2014[79]	United States	Prospective cohort study	Binary yes/no, Lysholm, SF-36, Alignment, ROM, Union, Pain	SPN	SPN midline	-	-	37	-	-	37	Yes	Yes	Yes	Yes	Yes	No	No	IV	-

			diagram, RUST score, Patellofemoral joint damage (arthroscopy)																	
Ryan 2014[80]	United States	Retrospective cohort study	Alignment, NRS - knee pain (0 - 3)	IPN vs SE	Semi-extended superomedial (IA)	IPN transpatellar		84	101	-	185	Yes	Yes	Yes	Yes	Yes	No	No	III	-
Jones 2014[81]	UK	Retrospective cohort study	Kujala, Radiation dose, Nail insertion location, SF-12, Accuracy of fracture reduction, Union, Shortening	IPN vs SPN	SPN midline	IPN medial parapatellar	-	30	29	-	59	No	Yes	Yes	No	No	No	No	III	23, 28
Chen 2014[82]		Retrospective cohort study	VAS - knee pain (0 - 10), Nail insertion location, VAS - rest (0 - 100), VAS - walking (0 - 100), VAS - running (0 - 100), VAS - squatting (0 - 100), VAS - kneeling (0 - 100), VAS - stair ascent (0 - 100), VAS - stair descent (0 - 100), Time to nail removal, Union, Nail prominence, Nail diameter	IPN	IPN transpatellar	-	-	108	-	-	108	Yes	Yes	No	Yes	No	No	Yes	III	26.8
Moreschini 2013[83, 84]	Italy	Retrospective cohort study	Radiation dose, Distal locking time	NR	No description	-	-	50	-	-	50	Yes	No	No	No	No	No	No	IV	-
Mir 2013	United States	Cadaveric	Nail insertion location, Principal strain	NA	NA	-	-	1	-	-	1	NA	NA	NA	NA	NA	NA	Yes	NA	-
Khan 2013[85]	Pakistan	Case series	Union, Hardware failure, Sikorski & Barrington scale, Hammer classification	NR	No description		-	50	-	-	50	No	No	Yes	No	No	No	No	IV	0.5, 1, 2, 3, 6, 9
Rothberg 2013[86]	United States	Case control study	Lysholm, Nail-apex distance, Union, Additional fixation	SE	Semi-extended lateral parapatellar (EA)	Semi-extended medial parapatellar (EA)	-	9	9	-	18	No	Yes	Yes	No	No	Yes	No	III	6, 12
Jankovic 2013[87]	Croatia	Retrospective cohort study	ROM, VAS - knee pain (1 - 10), Nail prominence, Saphenous nerve injury, Return to pre-injury level of activity, Tegner	IPN	IPN medial parapatellar	-	-	60	-	-	60	Yes	Yes	Yes	Yes	Yes	Yes	No	III	38.9
Gaines 2013[88]	United States	Cadaveric	Nail insertion location, Menisci damage (inspection post-dissection), Anterior cruciate	IPN vs SPN	SPN midline	IPN medial parapatellar	-	10	10	-	20	No	NA		Yes	NA	NA	No	NA	-

			ligament damage (inspection post-dissection), Intermeniscal ligament damage (inspection post-dissection), Articular surface damage (inspection post-dissection)																	
Bible 2013[89]	United States	Cadaveric	Nail insertion location, Surface area of cortical bone removed, Menisci damage (inspection post-dissection), Meniscotibial ligaments damage (inspection post-dissection), Articular surface damage (inspection post-dissection), Anterior cruciate ligament damage (inspection post-dissection), Intermeniscal ligament damage (inspection post-dissection)	IPN vs SPN	IPN medial parapatellar	SPN unspecified	-	18	18	-	36	NA	NA	NA	Yes	NA	NA	No	NA	NA
Aksahin 2013[90]	Turkey	Prospective cohort study	Kujala, Patellofemoral kinematics from CT, Rotational deformity	NR	No description	-	-	28	-	-	28	No	No	No	No	No	No	No	V	NR
Vaseenon 2012[91]	Thailand	Cadaveric	Nail insertion location, Articular surface damage	IPN	IPN transpatellar		-	12	-	-	12	Yes	NA	NA	No	NA	NA	No	NA	-
Theriault 2012[92]	Canada	Prospective cohort study	Rotational deformity, Lower Extremity Functional Scale, Olerud-Molander ankle score, Six-minute walk test	IPN	IPN transpatellar	-	-	70	-	-	70	No	No	No	No	No	No	No	II	58
Song 2012[93]	Republic of Korea	Retrospective cohort study	Nail prominence, VAS - rest (0 - 100), VAS - kneeling (0 - 100), VAS - squatting (0 - 100), VAS - long-term sitting (0 - 100), VAS - stair ascent (0 - 100), VAS - stair descent (0 - 100), Tegner, Modified	IPN	IPN medial parapatellar	-	-	45	-	-	45	Yes	Yes	Yes	Yes	No	Yes	No	IV	22.3

			Lysholm, Complications																
Schemitsch 2012[94]	Canada	Randomised controlled trial	Hardware exchange, Hardware removal, Infection, Bone grafting, Complications	NR	No description	-	-	1226	-	-	1226	Yes	Yes	Yes	Yes	Yes	Yes	No	II	12
Attal 2012[95]	Austria	Case series	Alignment, Complications, Implant failure, Infection, Reoperation, Union, Shortening, Rotational deformity	IPN	IPN unspecified	-	-	185	-	-	185	Yes	Yes	Yes	Yes	No	Yes	No	IV	3, 12
Leliveld 2012[96]	The Netherlands	Retrospective cohort study	Binary yes/no, SF-36, Anterior knee pain scale, Sensory function of infrapatellar nerve, Binary - kneeling	IPN	IPN medial parapatellar	IPN transpatellar	-	46	26	-	72	Yes	Yes	Yes	Yes	No	Yes	Yes	III	84
Herren 2012[97]	Germany	Case series	Oxford, ROM, Activities of daily living, Kneeling test, VAS - knee pain (ND)	SPN	SPN unspecified	-	-	26	-	-	26	No	No	No	No	No	No	No	IV	18
Cerqueira 2012[98]	Brazil	Cadaveric	Nail insertion location, Fat pad damage (inspection post-dissection), Patellar tendon damage (inspection post-dissection), Menisci damage (inspection post-dissection), Articular surface damage (inspection post-dissection)	SPN	SPN lateral	-	-	10	-	-	10	NA	NA	NA	NA	NA	NA	No	NA	-
Beltran 2012[99]	United States	Cadaveric	Nail insertion location, Fat pad damage (inspection post-dissection), Intermeniscal ligament damage (inspection post-dissection), Anterior cruciate ligament damage (inspection post-dissection), Menisci damage (inspection post-dissection)	SPN	SPN unspecified	-	-	15	-	-	15	NA	NA	NA	NA	NA	NA	No	NA	-
Walker 2011[100]	Canada	Cadaveric	Nail insertion location	NA	NA	-	-	12	-	-	12	No	NA	NA	No	NA	NA	No	NA	-

Ryan 2011[101]	United States	Retrospective cohort study	Binary yes/no, NRS - knee pain (0 - 3), Hammer classification	IPN	IPN medial parapatellar	-	-	443	-	-	443	No	No	Yes	No	Yes	Yes	No	III	0.5, 1.5, 4, 6, 9, 12
Labronici 2011[102]	Brazil	Retrospective cohort study	Binary yes/no, Nail insertion location, Nail prominence	NR	No description	-	-	30	-	-	30	Yes	No	No	Yes	No	No	Yes	IV	-
Freeman 2011[103]	United States	Cadaveric	Implant fatigue life	NR	No description	-	-	18	-	-	18	Yes	NA	NA	No	NA	NA	No	NA	-
Forman 2011[104]	United States	Retrospective cohort study	Alignment, Rotational deformity, Union	NR	No description	-	-	27	-	-	27	Yes	No	Yes	Yes	No	Yes	No	III	-
Darabos 2011[105]	Croatia	Prospective cohort study	VAS - knee pain (0 - 10), Nail insertion location	IPN	IPN medial parapatellar	-	-	50	-	-	50	Yes	No	No	Yes	Yes	No	Yes	II	12, 24, 36
Chakraborty 2011[106]	Nepal	Retrospective cohort study	Binary yes/no, Infection, Union	NR	No description	-	-	18	-	-	18	Yes	Yes	Yes	Yes	Yes	Yes	No	III	-
Sadeghpour 2011[107]	Iran	Randomised controlled trial	ROM, Nail prominence, VAS - knee pain (ND)	IPN	IPN transpatellar	IPN medial parapatellar	-	25	25	-	50	No	No	Yes	No	No	Yes	No	II	0.5, 1, 3, 6
Weninger 2010[108]	Austria	Cadaveric	Alignment	IPN	IPN medial parapatellar	-	-	32	-	-	32	Yes	Yes	NA	Yes	NA	NA	No	NA	-
Kubiak 2010[109]	United States	Case series	Complications	SE	Semi-extended lateral parapatellar (EA)	Semi-extended medial parapatellar (EA)	-	13	13	-	26	Yes	Yes	Yes	Yes	No	No	No	IV	-
Gelbke 2010[110]	United States	Cadaveric	Patellofemoral nail contact pressure, Patellofemoral nail contact force	IPN vs SPN	SPN midline	IPN medial parapatellar	-	8	9	-	17	No	NA	NA	No	NA	NA	No	NA	-
Eastman 2010[111]	United States	Cadaveric	Nail insertion location, Menisci damage (inspection post-dissection), Articular surface damage (inspection post-dissection), Anterior cruciate ligament damage (inspection post-dissection), Intermeniscal ligament damage (inspection post-dissection)	SPN	SPN midline	-	-	16	-	-	16	NA	NA	NA	Yes	NA	NA	No	NA	-
Eastman 2010[112]	United States	Cadaveric	Nail insertion location	SPN	SPN midline	-	-	16	-	-	16	NA	NA	NA	No	NA	NA	No	NA	-
Weninger 2009[113]	Austria	Cadaveric	Nail insertion location, Fat pad damage, Menisci damage, Anterior cruciate ligament damage, Intermeniscal ligament damage, Articular surface damage	IPN	IPN medial parapatellar	IPN transpatellar	IPN lateral parapatellar	6	6	6	18	No	NA	NA	No	NA	NA	No	NA	-
Weil 2009[114]	United States	Retrospective cohort study	Binary yes/no, Lysholm,	SE	Semi-extended	-	-	50	-	-	50	Yes	Yes	Yes	Yes	Yes	No	No	III	0.5, 1.5, 3,

			Alignment, ROM, Binary - kneeling, Nail prominence, Union		lateral parapatellar (EA)															6, 12, 24
Uzümcügil 2009[115]	Istanbul	Retrospective cohort study	Binary yes/no, Nail insertion location	IPN	IPN transpatellar	-	-	30	-	-	30	Yes	Yes	No	Yes	Yes	No	No	IV	45.2, 56.6
Lefaivre 2008[116]	Canada	Retrospective cohort study	SF-36, Alignment, ROM, Short Musculoskeletal Functional Assessment, NRS - kneeling (1 - 10), NRS - squatting (1 - 10), NRS - stair descent (1 - 10), NRS - stair ascent (1 - 10), NRS - long-term sitting (1 - 10), NRS - walking (1 - 10), NRS - running (1 - 10), NRS - rest (1 - 10), Binary - swelling after activity, Binary - swelling after prolonged standing, Venous status, Lower extremity neurological status, Nail prominence, Kellgren-Lawrence, Union, Ligamentous examination, Meniscal pathology testing, Signs of edema	NR	No description	-	-	56	-	-	56	Yes	Yes	Yes	Yes	No	No	No	III	168
Fanian 2008[117]	Iran	Prospective cohort study	VAS - knee pain (0 - 10), Binary - walking, Binary - kneeling, Binary - tailor position, Binary - stair ascent, Binary - stair descent, Binary - rest, Binary - use of Iranian water	IPN	IPN medial parapatellar	IPN transpatellar	-	87	145	-	232	Yes	No	Yes	No	No	No	No	IV	6
Bhandari 2008[118]	Canada	Randomised controlled trial	Implant exchange, Bone grafting, Complications, Union, Reoperation, Hardware failure	NR	No description	-	-	1226	-	-	1226	Yes	Yes	Yes	Yes	Yes	Yes	Yes	I	12
Väistö 2008[119]	Finland	Randomised controlled trial	Lysholm, Nail insertion location, VAS - rest (0 -	IPN	IPN transpatellar	IPN medial parapatellar	-	14	14	-	28	Yes	No	No	No	No	No	Yes	II	97.3

			100), VAS - walking (0 - 100), VAS - running (0 - 100), VAS - squatting (0 - 100), VAS - kneeling (0 - 100), VAS - stair climb (0 - 100), VAS - stair descent (0 - 100), VAS - long-term sitting (0 - 100), VAS - impairment caused by AKP (0 - 100), Iowa knee score, Tegner, Kannus score, Quadriceps strength, Hamstrings strength, Nail prominence																	
Giri 2008[120]	Nepal	Retrospective cohort study	Surgery time, Union, Complications, Shortening	IPN	IPN medial parapatellar	-	-	55	-	-	55	Yes	Yes	Yes	Yes	No	Yes	No	III	4
Väistö 2007[121]	Finland	Prospective cohort study	Lysholm, Quadriceps strength, Hamstrings strength, VAS - rest (0 - 100), VAS - walking (0 - 100), VAS - running (0 - 100), VAS - squatting (0 - 100), VAS - kneeling (0 - 100), VAS - stair ascent (0 - 100), VAS - stair descent (0 - 100), VAS - long-term sitting (0 - 100), Tegner, Iowa knee score	IPN	IPN transpatellar	IPN medial parapatellar	-	14	14	-	28	Yes	No	No	No	Yes	No	Yes	III	96
Cartwright-Terry 2007[122]	UK	Retrospective cohort study	VAS - knee pain (0 - 10), Alignment, Functional anterior knee pain score (0 - 50), Complications, Union, Rotational deformity, Return to work	IPN	IPN medial parapatellar	-	-	52	-	-	52	No	Yes	No	No	No	No	Yes	III	-
Vidyadhara 2006[123]	India	Case series	Binary yes/no, Alignment, ROM, Lower Extremity Functional scale, Time to partial weight-bearing, Infection,	SE	Semi-extended medial parapatellar (IA)		-	45	-	-	45	Yes	Yes	Yes	Yes	Yes	Yes	Yes	IV	1.5, 3, 6, 12

			Chondral damage, Complications, Union, Rotational deformity, Hardware failure, Bone grafting																	
Nork 2006[124]	Germany	Retrospective cohort study	Alignment, Union, Complications	IPN	IPN transpatellar	IPN lateral parapatellar	-	28	9	-	37	Yes	Yes	Yes	Yes	Yes	Yes	No	III	18.9
Bhattacharyya 2006[125]	United States	Retrospective cohort study	Binary yes/no, Lysholm, VAS - rest (0 - 100), VAS - kneeling (0 - 100), VAS - walking (0 - 100), VAS - running (0 - 100), Nail prominence, Combined VAS score (0 - 300)	IPN	IPN transpatellar, IPN medial parapatellar	-	-	70	-	-	70	Yes	No	Yes	Yes	No	No	No	II	20
Babis 2006[126]	Greece	Prospective cohort study	Binary yes/no, Surgery time, Radiation dose, ROM, Union, Complications	IPN	IPN transpatellar	-	-	115	-	-	115	Yes	No	Yes	Yes	Yes	Yes	Yes	II	32
Nork 2005[127]	United States	Retrospective cohort study	SF-36, Alignment, Musculoskeletal Functional Assessment, Reoperation, Union, Complications	NR	No description	-	-	36	-	-	36	Yes	Yes	Yes	Yes	Yes	Yes	No	IV	54
Fan 2005[128]	Taiwan	Prospective cohort study	Alignment, ROM, Union, Rotational deformity, Iowa ankle score	IPN	IPN medial parapatellar	-	-	20	-	-	20	Yes	Yes	Yes	Yes	Yes	Yes	No	III	6, 12, 18, 24
Audige 2005[129]	Switzerland	Retrospective cohort study	Surgery time, Union	NR	No description	-	-	181	-	-	181	No	Yes	Yes	Yes	No	Yes	No	III	15
Al Hussainy 2005[130]	UK	Case control study	Binary yes/no, Surgery time, Nail insertion location, Union, Binary - squatting, Binary - kneeling, Binary - climbing, Binary - walking, Binary - rest	IPN	IPN transpatellar	IPN medial parapatellar	-	26	18	-	44	No	Yes	Yes	Yes	No	Yes	No	III	25
Väistö 2005[131]	Finland	Randomised controlled trial	Patellar tendon ultrasound examination, VAS - walking (0 - 100), VAS - running (0 - 100), VAS - squatting (0 - 100), VAS - kneeling (0 - 100), VAS - stair descent (0 - 100), VAS - stair climb (0 - 100), VAS - long-term sitting (0 - 100), VAS - rest (0 - 100)	IPN	IPN transpatellar	IPN medial parapatellar	-	20	16	-	36	Yes	No	No	Yes	Yes	No	Yes	II	30.02

Väistö 2004[132]	Finland	Prospective cohort study	Lysholm, Nail insertion location, Hamstrings strength, Quadriceps strength, VAS - rest (0 - 100), VAS - walking (0 - 100), VAS - running (0 - 100), VAS - squatting (0 - 100), VAS - kneeling (0 - 100), VAS - stair ascent (0 - 100), VAS - stair descent (0 - 100), VAS - long-term sitting (0 - 100), Tegner, Iowa knee score, Complications	IPN	IPN transpatellar	IPN medial parapatellar	-	20	20	-	40	Yes	No	No	Yes	Yes	No	Yes	III	38.4
Puloski 2004[133]	Canada	Prospective cohort study	Rotational deformity	NR	No description	-	-	22	-	-	22	Yes	Yes	Yes	Yes	No	No	No	III	-
Laflamme 2003[134]	Canada	Cadaveric	Biomechanical construct stability	NA	No description	-	-	20	-	-	20	Yes	NA	NA	Yes	NA	NA	No	NA	-
Samuelson 2002[135]	United States	Cadaveric	Nail insertion location	NA	NA	-	-	57	-	-	57	NA	NA	NA	No	NA	NA	No	NA	-
Kahn 2002[136]	United States	Case report	Rotational deformity, Shortening	NR	No description	-	-	3	-	-	3	Yes	No	Yes	Yes	No	Yes	Yes	V	-
Gorczyca 2002[137]	United States	Cadaveric	Compression stiffness, Torsional stiffness, Peak compression-bending stiffness	NR	No description	-	-	10	-	-	10	Yes	NA	NA	NA	NA	NA	No	NA	-
Dogra 2002[138]	UK	Retrospective cohort study	VAS - knee pain (0 - 10), SF-36, Union, VAS - fracture site (0 - 10), Iowa knee score, Hardware failure, Complications	IPN	IPN transpatellar	IPN medial parapatellar	-	75	7	-	83	No	No	Yes	Yes	Yes	Yes	Yes	III	36
Toivanen 2002[139]	Finland	Randomised controlled trial	Lysholm, ROM, VAS - rest (0 - 100), VAS - walking (0 - 100), VAS - running (0 - 100), VAS - squatting (0 - 100), VAS - kneeling (0 - 100), VAS - stair climb (0 - 100), VAS - stair descent (0 - 100), VAS - long-term sitting (0 - 100), Iowa knee score, Tegner, Nail prominence, Quadriceps	IPN	IPN transpatellar	IPN medial parapatellar	-	21	21	-	42	Yes	No	Yes	No	No	No	Yes	I	20.4

			strength, Hamstrings strength, Kannus score																	
Ricci 2001[140]	United States	Prospective cohort study	Alignment, Union, Complications	IPN	IPN medial parapatellar	-	-	12	-	-	12	Yes	Yes	Yes	No	No	Yes	No	IV	-
Nyland 2001[141]	United States	Retrospective cohort study	NRS - chair rise (1 - 10), NRS - unlevel walking (1 - 10), NRS - level walking (1 - 10), NRS - pivoting (1 - 10), NRS - squatting (1 - 10), NRS - stairs (1 - 10), NRS - catching (1 - 10), NRS - swelling (1 - 10), NRS - buckling (1 - 10), NRS - stiffness (1 - 10), NRS - knee pain (1 - 10), Hamstrings strength, Quadriceps strength	IPN	IPN medial parapatellar	-	-	10	-	-	10	No	No	Yes	No	No	Yes	No	IV	18
McConnell 2001[142]	United States	Cadaveric	Nail insertion location	IPN	NA	-	-	20	-	-	20	No	NA	NA		NA	NA	No	NA	-
Lembcke 2001[143]	Germany	Cadaveric	Nail insertion location, Alignment, Shortening	IPN	IPN medial parapatellar	IPN lateral parapatellar	-	11	11	-	22	No	NA	NA	No	NA	NA	No	NA	-
Tyllianakis 2000[144]	Greece	Retrospective cohort study	Binary yes/no, Alignment, ROM, Union, Shortening, Return to work, Rotational deformity, Hardware failure, Return to sport/exercise, Binary - walking	NR	No description		-	73	-	-	73	Yes	Yes	Yes	Yes	Yes	No	No	IV	34.2
Karachalios 2000[145]	Greece	Prospective cohort study	Binary yes/no, Surgery time, Radiation dose, Nail insertion location, Alignment, ROM, Union, Infection, Return to work, Hardware failure, Shortening, Rotational deformity	IPN	IPN transpatellar	-	-	60	-	-	60	Yes	Yes	Yes	Yes	Yes	Yes	No	II	5
Hernigou 2000[146]	France	Cadaveric	Nail insertion location, Menisci damage, Articular surface damage, Intermeniscal ligament damage	IPN	NA	-	-	54	-	-	54	NA	NA	NA	No	NA	NA	No	NA	-
Finkemeier 2000[147]	United States	Prospective cohort study	Union, Hardware failure, Infection,	NR	No description	-	-	94	-	-	94	Yes	Yes	Yes	Yes	Yes	Yes	No	III	19

			Compartment syndrome, Complications																	
Dogra 2000[148]	UK	Retrospective cohort study	Binary yes/no, VAS - knee pain (0 - 10), Alignment, Return to work, Iowa knee score, Shortening, Rotational deformity, Union, Time to full weight-bearing	IPN	IPN transpatellar	-	-	15	-	-	15	No	Yes	Yes	Yes	Yes	No	Yes	IV	56.4
Krettek 1999[149]	Germany	Prospective cohort study	Surgery time, Alignment, Union, Complications, Karström-Olerud score, Hardware failure	NR	No description	-	-	21	-	-	21	Yes	Yes	Yes	No	Yes	No	No	III	-
Tornetta 1999[150]	United States	Cadaveric	Nail insertion location, Menisci damage (inspection post-dissection), Anterior cruciate ligament damage (inspection post-dissection), Articular surface damage (inspection post-dissection)	IPN	IPN medial parapatellar	IPN lateral parapatellar	-	20	20	-	40	NA	NA	NA	Yes	NA	NA	No	NA	-
Moed 1998[151]	United States	Prospective cohort study	Hardware failure, Ultrasound examination of fracture site, Reoperation, Union	NR	No description		-	50	-	-	50	Yes	Yes	Yes	Yes	Yes	Yes	Yes	III	1.5, 2.75
Lovell 1998[152]	UK	Retrospective cohort study	Binary yes/no, Patella-tendon shortening	IPN	IPN medial parapatellar	IPN transpatellar	-	22	16	-	38	No	No	Yes	Yes	No	No	No	IV	6
Krettek 1998[153]	United States	Cadaveric	Surgery time, Radiation dose, Distal locking time, Screw wear, Hardware failure	NA	NA	-	-	20	-	-	20	Yes	NA	NA	Yes	NA	NA	No	NA	-
Devitt 1998[154]	UK	Cadaveric	Patellofemoral joint force, Patellofemoral joint pressure	IPN	IPN medial parapatellar	IPN transpatellar	-	8	8	-	16	NA	NA	NA	Yes	NA	NA	No	NA	-
Hallam 1998[155]	UK	Retrospective cohort study	Blackburne-Peel index	IPN	IPN transpatellar	IPN medial parapatellar	-	9	9	-	18	No	No	No	No	No	No	No	III	-
Tornetta 1997[156]	United States	Prospective cohort study	Anterior compartment pressure, Compartment syndrome	NR	No description	-	-	59	-	-	59	No	No	No	Yes	Yes	Yes	No	III	-
Keating 1997[157]	Canada	Retrospective cohort study	Binary yes/no, Nail insertion location, Alignment, Knee pain time of onset,	IPN	IPN medial parapatellar	IPN transpatellar	No description	65	36	9	110	Yes	No	Yes	Yes	Yes	Yes	Yes	III	32

			Complications, Union, Shortening, Rotational deformity, Nail prominence, Binary - activity, Pain exacerbated by kneeling, Infection, Compartment syndrome, Bone grafting, Revision nailing, Return to sport/exercise, Pain influence on leisure activity																	
Court-Brown 1997[158]	UK	Retrospective cohort study	VAS - knee pain (0 - 10), VAS - kneeling (0 - 10), VAS - squatting (0 - 10), VAS - running (0 - 10), VAS - walking (0 - 10), VAS - jumping (0 - 10), VAS - stair ascent (0 - 10), VAS - ladder ascent (0 - 10), VAS - rest (0 - 10), Complications	IPN	IPN transpatellar	IPN medial parapatellar	-	62	97	-	159	Yes	Yes	Yes	Yes	No	No	No	III	-
Buehler 1997[159]	United States	Case series	Alignment, Union, Complications	IPN	IPN transpatellar, IPN lateral parapatellar	-	-	14	-	-	14	Yes	No	Yes	No	No	Yes	No	IV	6
Blachut 1997[160]	Canada	Randomised controlled trial	Surgery time, Blood loss, Radiation dose, Alignment, Union, Implant failure, Infection, Complications, Reoperation, Shortening, Rotational deformity	IPN	IPN transpatellar, IPN medial parapatellar	-	-	136	-	-	136	Yes	Yes	Yes	Yes	Yes	Yes	No	I	12
Kneifel 1996[161]	Buckley	Randomised controlled trial	Complications, Union	IPN	IPN medial parapatellar	-	-	42	-	-	42	Yes	No	Yes	Yes	Yes	No	No	I	-
Court-Brown 1996[162]	UK	Randomised controlled trial	Binary yes/no, ROM, Union, Infection, Reoperation, Hardware failure, Return to work, Binary - awkward positions, Binary - kneeling, Binary - stooping/crawling, Binary - long walks, Binary - stairs, Binary - walking on	IPN	IPN medial parapatellar	-	-	50	-	-	50	Yes	Yes	Yes	Yes	Yes	Yes	Yes	I	3, 6, 12

			difficult ground, Binary - jumping, Binary - climbing																	
Boenisch 1996[163]	UK	Case series	Alignment, ROM, Union, Shortening, Rotational deformity, Locking bolt failure, Infection, Hospital stay, Complications	NR	No description	-	-	72	-	-	72	Yes	Yes	Yes	Yes	Yes	Yes	No	IV	15.2
Tornetta 1996[164]	United States	Case series	Alignment, Complications	SE	Semi-extended medial parapatellar (IA)	-	-	30	-	-	30	Yes	Yes	Yes	Yes	No	No	No	IV	-
Orfaly 1995[165]	Canada	Retrospective cohort study	Binary yes/no, Nail insertion location, Nail prominence, Hardware removal	IPN	IPN medial parapatellar	IPN transpatellar	-	65	36	-	110	Yes	No	Yes	Yes	No	No	Yes	IV	16
Lang 1995[166]	United States	Retrospective cohort study	Alignment, Union, Exchange nailing, Shortening	IPN	IPN medial parapatellar	IPN lateral parapatellar	IPN transpatellar	30	1	1	32	Yes	No	Yes	Yes	No	Yes	Yes	III	25
Gregory 1995[167]	United States	Case series	Binary yes/no, ROM, Union, Binary - stairs, Return to work, Return to sport/exercise, Johner & Wruhs criteria	NR	No description	-	-	38	-	-	38	Yes	No	Yes	Yes	Yes	Yes	No	IV	26
Freedman 1995[168]	United States	Retrospective cohort study	Nail insertion location, Alignment, Union	NR	No description	-	-	133	-	-	133	Yes	Yes	No	Yes	No	Yes	No	IV	-
Anglen 1995[169]	United States	Retrospective cohort study	Binary yes/no, Surgery time, Blood loss, Alignment, Union, Complications	IPN	IPN medial parapatellar	-	-	38	-	-	38	Yes	Yes	Yes	Yes	Yes	Yes	Yes	IV	11.5
O'Dwyer 1994[170]	UK	Prospective cohort study	Union, Complications, Infection	IPN	IPN medial parapatellar	-	-	70	-	-	70	Yes	Yes	Yes	Yes	No	Yes	No	II	
Habernek 1992[171]	Austria	Retrospective cohort study	Binary yes/no, Alignment, ROM, Complications, Sick leave, Johner & Wruhs criteria, Compartment syndrome, Infection, Binary - long walks, Binary - pain after change of weather, Return to sport/exercise	IPN	No description	No description	No description	92	-	-	92	Yes	Yes	Yes	No	Yes	No	Yes	IV	33
Koval 1991[172]	United States	Retrospective cohort study	Alignment, ROM, Union, Complications	IPN	IPN transpatellar	IPN medial parapatellar	-	42	18	-	60	Yes	Yes	Yes	Yes	Yes	Yes	Yes	III	25

Carr 1991[173]	United States	Cadaveric, Retrospective cohort study	Binary yes/no, Alignment, Bursting strain, Complications	NR	No description	No description	-	8	27	-	8, 27	No	Yes	No	No	No	No	No	NA, V	6, 36
Court-Brown 1990[174]	UK	Retrospective cohort study	Binary yes/no, Surgery time, Radiation dose, ROM, Union, Hospital stay, Hardware removal, Complications, Return to work, Return to sport/exercise, Shortening, Infection, Compartment syndrome, Nerve damage, Rotational deformity	IPN	IPN medial parapatellar	-	-	125	-	-	125	Yes	Yes	Yes	Yes	Yes	Yes	Yes	III	3, 6, 12, 20
Alho 1990[175]	Norway	Case series	Binary yes/no, Surgery time, Alignment, Union, Complications, Return to work, ROM, Shortening, Rotational deformity	IPN	IPN unspecified	-	-	93	-	-	93	Yes	Yes	Yes	Yes	Yes	Yes	No	IV	22
Ekeland 1988[176]	Norway	Case series	Surgery time, Alignment, ROM, Union, Time to full weight-bearing, Shortening, Return to work, Change in working capacity, Return to sport/exercise	IPN	IPN transpatellar	-	-	45	-	-	45	Yes	Yes	Yes	Yes	Yes	Yes	Yes	IV	16
Bone 1986[177]	United States	Retrospective cohort study	Alignment, ROM, Union, Infection, Shortening	NR	No description	-	-	100	-	-	100	Yes	Yes	Yes	Yes	Yes	Yes	Yes	III	15
Donald 1983[178]	United States	Case series	Surgery time, Radiation dose, Alignment, Union, Return to work, Shortening, Complications	IPN	IPN transpatellar	-	-	50	-	-	50	No	No	No	Yes	Yes	Yes	Yes	IV	12
Hamza 1971[179]	UK	Retrospective cohort study	Union, Return to work, Authors' assessment, Complications	IPN	IPN transpatellar	-	-	50	-	-	50	No	Yes	Yes	Yes	Yes	No	No	IV	6, 42

Abbreviations:

1. Sahni G, Singh S, Kavia A, Aggarwal HO, Chawla HKS. Suprapatellar versus Infrapatellar Approach for Intramedullary Nailing in Tibial Shaft Fractures: A Prospective Interventional Study. Journal of Clinical and Diagnostic Research. 2023;17(1):RC01-RC4.

2. Gupta N, Sharma SK, Basit A, Gupta S, Anjum R, Deep A, et al. Suprapatellar Versus Infrapatellar Nail In Distal Tibial Fractures. European Journal of Molecular & Clinical Medicine. 2023;10(1):2575-80.
3. Chahal JS, Lal M, Goel D, Gautam D. Functional Outcome Of Suprapatellar And Infrapatellar Intramedullary Nailing For Extra Articular Tibia Fractures: A Comparative Study. NeuroQuantology. 2022;20(17):1338.
4. Lu K, Wu Z-q, Wang H-z, Qian R-x, Li C, Gao Y-j. The semi-extended infrapatellar intramedullary nailing of distal tibia fractures: a randomized clinical trial. Journal of orthopaedics and traumatology. 2022;23(1):53-.
5. Leliveld MS, Van Lieshout EMM, Polinder S, Verhofstad MHJ, the TSI. Effect of Transverse Versus Longitudinal Incisions on Anterior Knee Pain After Tibial Nailing (TRAVEL): A Multicenter Randomized Trial with 1-Year Follow-up. JBJS. 2022;104(24).
6. Basile A, Palmieri L, Lanzetti R, Sessa P, Spoliti M, Giai Via A, et al. Suprapatellar intramedullary nailing of tibial shaft fractures in pregnancy. A report of two cases. BMC Pregnancy and Childbirth. 2022;22(1) (no pagination)(528).
7. He M, Liu J, Deng X. Controlling the angle between the distal locking screw and tibiotalar joint tangent helps to reduce the occurrence of misalignment of distal tibial fractures treated with intramedullary nail fixation. BMC musculoskeletal disorders. 2022;23(1) (no pagination)(671).
8. Sathy A, Prabhakar P, Harirah M, Collett G, Nakonezny P. Low rate of malalignment using the tibial traction triangle for infrapatellar nailing of distal tibia fractures. Injury. 2022;53(4):1539-42.
9. Yasuda T, Sato K, Yamazaki K, Arai M, Shinohara D, Taisuke Y, et al. Nail insertion points in semi-extended nailing of tibial fractures and their influence on alignment: A retrospective cohort study comparing two nail insertion techniques. Injury. 2022;53(10):3508-16.
10. Approach S, Gurumoorthy M, Singh D, Manikandarajan A, Dharani S. Functional and Radiological Outcome of Proximal 1/3rd Tibial Fractures Managed with Intramedullary Through. European Journal of Molecular and Clinical Medicine. 2022;9(1):12-24.
11. Gao F, Wang XH, Xia SL, Zhou XX, Wang MH, Fu BG, et al. Intramedullary Nail Fixation by Suprapatellar and Infrapatellar Approaches for Treatment of Distal Tibial Fractures. Orthop Surg. 2022;14(9):2350-60.
12. Llano L, Soruco ML, Taype Zamboni D, Sancineto C, Barla J, Carabelli G. Comparison between functionality and quality of reduction between suprapatellar vs infrapatellar approaches in the treatment of diaphyseal and distal tibial fractures. European journal of orthopaedic surgery & traumatology : orthopedie traumatologie. 2022.
13. Patel AH, Wilder JH, Lee OC, Ross AJ, Vemulapalli KC, Gladden PB, et al. A Review of Proximal Tibia Entry Points for Intramedullary Nailing and Validation of The Lateral Parapatellar Approach as Extra-articular. Orthopedic reviews. 2022;14(1).
14. Umur L, Sari E, Orhan S, Sürücü S, Yildirim C. Dilemma of Supra- or Infrapatellar Tibial Nailing: Anterior Knee Pain vs. Intra-Articular Damage. Int J Clin Pract. 2022;2022:8220030.
15. Al-Azzawi M, Davenport D, Shah Z, Khakha R, Afsharpad A. Suprapatellar versus infrapatellar nailing for tibial shaft fractures: A comparison of surgical and clinical outcomes between two approaches. Journal of Clinical Orthopaedics and Trauma. 2021;17:1-4.
16. Baker HP, Strelzow J, Dillman D. Tibial alignment following intramedullary nailing via three approaches. European journal of orthopaedic surgery & traumatology : orthopedie traumatologie. 2021.
17. Daley-Lindo TS, Kerr M, Haidukewych GJ, Koval KJ, Parry JA, Langford JR. Long-Term Patient-Reported Knee Outcomes After Suprapatellar Intramedullary Tibial Nailing. Indian journal of orthopaedics. 2021;55(3):669-72.
18. Fontalis A, Weil S, Williamson M, Houston J, Ads T, Trompeter A. A comparison of anterior knee pain, kneeling pain and functional outcomes in suprapatellar versus infrapatellar tibial nailing. European Journal of Orthopaedic Surgery and Traumatology. 2021;31(6):1143-50.
19. Graulich T, Gerhardy J, Omar Pacha T, Örgel M, Macke C, Krettek C, et al. Patella baja after intramedullary nailing of tibial fractures, using an infrapatellar/transtendinous approach, predicts worse patient reported outcome. European journal of trauma and emergency surgery : official publication of the European Trauma Society. 2021.

20. Hague M, Texeira D, Anderson T, Williamson M, Trompeter A. Nailing distal tibial fractures: does entry technique affect distal alignment? European journal of orthopaedic surgery & traumatology : orthopedie traumatologie. 2021.
21. Khan MN, Hafeez A, Faraz A, Naveed E, Ilyas MW, Rasool MU, et al. Comparison of Medial Parapatellar and Transpatellar Tendon Approach in Intramedullary Interlocking Nailing for Tibial Fracture: A Retrospective Analysis. Cureus. 2021;13(8):e17404.
22. Lu K, Gao YJ, Li C, Wu ZQ, Yin Y, Wang HZ. Semi-extended intramedullary nailing of the tibia using an infrapatellar approach: a retrospective cohort study. International orthopaedics. 2021;45(10):2719-26.
23. Metcalf KB, Du JY, Lapite IO, Wetzel RJ, Sontich JK, Dachenhaus ER, et al. Comparison of Infrapatellar and Suprapatellar Approaches for Intramedullary Nail Fixation of Tibia Fractures. Journal of orthopaedic trauma. 2021;35(2):e45-e50.
24. Shi GG, Kumar AR, Ledford CK, Ortiguera CJ, Wilke BK. Infrapatellar Saphenous Nerve Is at Risk During Tibial Nailing: An Anatomic Study. Journal of the American Academy of Orthopaedic Surgeons Global research & reviews. 2021;5(10).
25. Zhao J, Qu L, Li P, Tan C, Tao C. Lateral position: a friendly surgical position for intramedullary nailing of tibial shaft fractures via infrapatellar approach. BMC musculoskeletal disorders. 2021;22(1).
26. Zhu Z, Wang Z, Zhou P, Wang X, Guan J. Comparison of clinical efficacy of suprapatellar and infrapatellar intramedullary nailing in treating tibial shaft fractures. Pakistan Journal of Medical Sciences. 2021;37(7):1753-7.
27. Xu L, Zhu W, Xie K, Liu L, Zhang X, Yang J, et al. Tibial intramedullary nailing in the lateral decubitus position: Technical notes and preliminary clinical outcomes. Medicine. 2020;99(28):e21234.
28. Singh AK, Sait S, Khan Y, Al-Obaidi B, Bhattacharya R. Supra-patellar nailing for isolated closed tibial shaft fractures: Medium term functional outcomes from an Academic Level 1 Trauma centre. Injury. 2020;51(7):1642-6.
29. Lu Y, Wang G, Hu B, Ren C, Sun L, Wang Z, et al. Comparison of suprapatellar versus infrapatellar approaches of intramedullary nailing for distal tibia fractures. Journal of orthopaedic surgery and research. 2020;15(1):422.
30. Lu K, Gao YJ, Wang HZ, Li C, Qian RX, Dong QR. Comparison between infrapatellar and suprapatellar approaches for intramedullary nailing for the fractures of the tibial shaft. European journal of trauma and emergency surgery : official publication of the European Trauma Society. 2020.
31. Kulkarni MS, Tummala M, Aroor MN, Vijayan S, Rao SK. Suprapatellar nailing in proximal third tibial fractures - Clinicoradiological outcome. Injury. 2020;51(8):1879-86.
32. Cinats DJ, Viskontas D, Boyer D, Perey B, Stone T. The Influence of Sagittal Proximal Tibial Anatomy in Tibial Intramedullary Nailing. Journal of orthopaedic trauma. 2020;34(11):606-11.
33. Allen JD, Matuszewski PE, Comadoll SM, Hamilton DA, Abbenhaus EJ, Aneja A, et al. The Learning Curve of Suprapatellar Nailing: Adoption Over Time Can Decrease Operative Time and Radiation Exposure. Journal of orthopaedic trauma. 2020;34(7):370-5.
34. Ozcan C, Turkmen I, Sokucu S. Comparison of three different approaches for anterior knee pain after tibia intramedullary nailing. European journal of trauma and emergency surgery : official publication of the European Trauma Society. 2020;46(1):99-105.
35. Maslow JI, Joseph HL, Hong DY, Henry AL, Mitchell PM, Collinge CA. Radiographic Evaluation of the Tibial Intramedullary Nail Entry Point. The Journal of the American Academy of Orthopaedic Surgeons. 2020.
36. Lu K, Gao YJ, Wang HZ, Li C, Zhou TT, Qian RX, et al. A comparison of the use of a suprapatellar Chinese Aircraft-shaped Sleeve System versus suprapatellar intramedullary nailing for tibial shaft fractures: Outcomes over a one-year follow-up. Injury. 2020.
37. Hessmann MH, Buhl M, Finkemeier C, Khoury A, Mosheiff R, Blauth M. Suprapatellar nailing of fractures of the tibia. Operative Orthopadie und Traumatologie. 2020.
38. Valsamis EM, Iliopoulos E, Williams R, Trompeter A. Suprapatellar tibial nailing: a learning curve analysis. European journal of trauma and emergency surgery : official publication of the European Trauma Society. 2020;46(5):1107-13.

39. Stella M, Santolini E, Felli L, Santolini F, Horwitz DS. Semiextended Tibial Nail Insertion Using an Extraarticular Lateral Parapatellar Approach: A 24-Month Follow-up Prospective Cohort Study. Journal of orthopaedic trauma. 2019;33(10):e366-e71.
40. Serbest S, Tiftikçi U, Çoban M, Çirpar M, Dağlar B. Knee Pain and Functional Scores after Intramedullary Nailing of Tibial Shaft Fractures Using a Suprapatellar Approach. Journal of orthopaedic trauma. 2019;33(1):37-41.
41. Rothberg DL, Stuart AR, Presson AP, Haller JM, Higgins TF, Kubiak EN. A Comparison of the Open Semi-extended Parapatellar Versus Standard Entry Tibial Nailing Techniques and Knee Pain: A Randomized Controlled Trial. Journal of orthopaedic trauma. 2019;33(1):31-6.
42. Özbek EA, Kalem M, Kinik H. Do the loss of thigh muscle strength and tibial malrotation cause anterior knee pain after tibia intramedullary nailing? BioMed research international. 2019;2019.
43. Nicolescu R, Quinnan SM, Lawrie CM, Hutson JJ. Tensioned Wire-Assisted Intramedullary Nail Treatment of Proximal Tibia Shaft Fractures: A Technical Trick. Journal of orthopaedic trauma. 2019;33(3):e104-e9.
44. MacDonald DRW, Caba-Doussoux P, Carnegie CA, Escriba I, Forward DP, Graf M, et al. Tibial nailing using a suprapatellar rather than an infrapatellar approach significantly reduces anterior knee pain postoperatively: a multicentre clinical trial. The bone & joint journal. 2019;101-b(9):1138-43.
45. Ladurner A, Acklin YP, Mueller TS, Sommer C. Decrease surgery time by using an alternative lateral parapatellar approach for tibia shaft fracture nailing. Archives of orthopaedic and trauma surgery. 2019;139(7):943-9.
46. Isaac M, O'Toole RV, Udogwu U, Connelly D, Baker M, Lebrun CT, et al. Incidence of Knee Pain Beyond 1 Year: Suprapatellar Versus Infrapatellar Approach for Intramedullary Nailing of the Tibia. Journal of orthopaedic trauma. 2019;33(9):438-42.
47. Cui Y, Hua X, Schmidutz F, Zhou J, Yin Z, Yan SG. Suprapatellar versus infrapatellar approaches in the treatment of tibia intramedullary nailing: a retrospective cohort study. BMC musculoskeletal disorders. 2019;20(1):573.
48. Çiçekli Ö, Topçu HN, Kochai A, Şükür E, Türker M. Comparison of suprapatellar and infrapatellar tibial nailing: More anatomic entry point and fracture reduction via the suprapatellar approach. International Journal of Clinical and Experimental Medicine. 2019;12(1):804-11.
49. Çiçekli Ö, Kochai A, Şükür E, Başak AM, Kurtoğlu A, Türker M. Suprapatellar approach for fractures of the tibia: Does the fracture level matter? Eklem hastaliklari ve cerrahisi = Joint diseases & related surgery. 2019;30(1):10-6.
50. Bhakta P, Reazaul Karim HM, O'Brien B, Vassallo MC, Leliveld MS, Kamphuis SJM, et al. An infrapatellar nerve block reduces knee pain in patients with chronic anterior knee pain after tibial nailing: a randomized, placebo-controlled trial in 34 patients. Acta orthopaedica. 2019;90(5):505-6.
51. Anderson TRE, Beak PA, Trompeter AJ. Intra-medullary nail insertion accuracy: A comparison of the infra-patellar and supra-patellar approach. Injury. 2019;50(2):484-8.
52. Williamson M, Iliopoulos E, Williams R, Trompeter A. Intra-operative fluoroscopy time and radiation dose during suprapatellar tibial nailing versus infrapatellar tibial nailing. Injury. 2018;49(10):1891-4.
53. Tajima K, Shimizu C, Ohno S, Nishida Y, Udagawa K, Sasaki J. A Heart-shaped Sleeve Simplifies Intramedullary Tibial Nail Insertion when Using the Suprapatellar Approach. The Keio journal of medicine. 2018;67(1):10-6.
54. Schumaier AP, Avilucea FR, Southam BR, Sinha P, Le TT, Wyrick JD, et al. Terminal position of a tibial intramedullary nail: a computed tomography (CT) based study. European journal of trauma and emergency surgery : official publication of the European Trauma Society. 2018.
55. Lu K, Zhou TT, Gao YJ, Wang HZ, Wu ZQ, Wang Y, et al. Application of the Chinese Aircraft-shaped Sleeve system in the treatment of tibial shaft fractures using a suprapatellar approach for tibial intramedullary nailing: a randomised controlled trial. Journal of orthopaedic surgery and research. 2018;13(1):286.
56. Franke J, Homeier A, Metz L, Wedel T, Alt V, Spat S, et al. Infrapatellar vs. suprapatellar approach to obtain an optimal insertion angle for intramedullary nailing of tibial fractures. Eur J Trauma Emerg Surg. 2018;44(6):927-38.
57. Cazzato G, Saccomanno MF, Noia G, Masci G, Peruzzi M, Marinangeli M, et al. Intramedullary nailing of tibial shaft fractures in the semi-extended position using a suprapatellar approach: A retrospective case series. Injury. 2018;49:S61-S4.

58. Triantafillou K, Barcak E, Villarreal A, Collinge C, Perez E. Proper Distal Placement of Tibial Nail Improves Rate of Malalignment for Distal Tibia Fractures. Journal of orthopaedic trauma. 2017;31(12):e407-e11.
59. Turkmen I, Saglam Y, Turkmensoy F, Kemah B, Kara A, Unay K. Influence of sagittal plane malpositioning of the patella on anterior knee pain after tibia intramedullary nailing. European journal of orthopaedic surgery & traumatology : orthopedie traumatologie. 2017;27(1):133-9.
60. Mehta N, Selvaratnam V, Giotakis N, Narayan B. Is device-assisted reduction prior to semi-extended intramedullary nailing of distal tibial fractures necessary? Injury. 2017;48(2):506-10.
61. Soraganvi PP, Anand-Kumar BB, Rajagopalakrishnan RR, Praveen-Kumar BB. Anterior Knee Pain after Tibial Intra-medullary Nailing: Is it Predictable? Malaysian orthopaedic journal. 2016;10(2):16-20.
62. De Giacomo AF, Tornetta rP. Alignment After Intramedullary Nailing of Distal Tibia Fractures Without Fibula Fixation. Journal of orthopaedic trauma. 2016;30(10):561-7.
63. Zamora R, Wright C, Short A, Seligson D. Comparison between suprapatellar and parapatellar approaches for intramedullary nailing of the tibia. Cadaveric study. Injury. 2016;47(10):2087-90.
64. Sun Q, Nie XY, Gong JP, Wu JZ, Li RL, Ge W, et al. The outcome comparison of the suprapatellar approach and infrapatellar approach for tibia intramedullary nailing. International orthopaedics. 2016;40(12):2611-7.
65. Obremskey W, Agel J, Archer K, To P, Tornetta P, 3rd. Character, Incidence, and Predictors of Knee Pain and Activity After Infrapatellar Intramedullary Nailing of an Isolated Tibia Fracture. J Orthop Trauma. 2016;30(3):135-41.
66. Fu B. Locked META intramedullary nailing fixation for tibial fractures via a suprapatellar approach. Indian Journal of Orthopaedics. 2016;50(3):283-9.
67. Chan DS, Serrano-Riera R, Griffing R, Steverson B, Infante A, Watson D, et al. Suprapatellar versus infrapatellar tibial nail insertion: a prospective randomized control pilot study. J Orthop Trauma. 2016;30(3):130-4.
68. Bakhsh WR, Cherney SM, McAndrew CM, Ricci WM, Gardner MJ. Surgical approaches to intramedullary nailing of the tibia: Comparative analysis of knee pain and functional outcomes. Injury. 2016;47(4):958-61.
69. Avilucea FR, Triantafillou K, Whiting PS, Perez EA, Mir HR. Suprapatellar Intramedullary Nail Technique Lowers Rate of Malalignment of Distal Tibia Fractures. Journal of orthopaedic trauma. 2016;30(10):557-60.
70. Aksahin E, Yilmaz S, Karasoy I, Duran S, Yuksel HY, Dogan O, et al. Sagittal patellar tilt and concomitant quadriceps hypotrophy after tibial nailing. Knee surgery, sports traumatology, arthroscopy : official journal of the ESSKA. 2016;24(9):2878-83.
71. Ahmad S, Ahmed A, Khan L, Javed S, Ahmed N, Aziz A. Comparative Analysis Of Anterior Knee Pain In Transpatellar And Medial Parapatellar Tendon Approaches In Tibial Interlocking Nailing. Journal of Ayub Medical College, Abbottabad : JAMC. 2016;28(4):694-7.
72. LaPrade MD, LaPrade CM, Hamming MG, Ellman MB, Turnbull TL, Rasmussen MT, et al. Intramedullary Tibial Nailing Reduces the Attachment Area and Ultimate Load of the Anterior Medial Meniscal Root: A Potential Explanation for Anterior Knee Pain in Female Patients and Smaller Patients. The American journal of sports medicine. 2015;43(7):1670-5.
73. Kruppa CG, Hoffmann MF, Sietsema DL, Mulder MB, Jones CB. Outcomes After Intramedullary Nailing of Distal Tibial Fractures. Journal of orthopaedic trauma. 2015;29(9):e309-e15.
74. Courtney PM, Boniello A, Donegan D, Ahn J, Mehta S. Functional knee outcomes in infrapatellar and suprapatellar tibial nailing: does approach matter? American journal of orthopedics (Belle Mead, NJ). 2015;44(12):E513-6.
75. Tonk G, Menwal G, Kumar Gupta A, Rustagi N, Kumar A. ANTERIOR KNEE PAIN IN TRANSTENDINOUS AND PARATENDINOUS APPROACHES OF TIBIAL INTERLOCKING NAIL: A COMPARATIVE STUDY. Journal of evolution of medical and dental sciences. 2014;3(28):7874-80.
76. Say F, Bülbül M. Findings related to rotational malalignment in tibial fractures treated with reamed intramedullary nailing. Archives of orthopaedic and trauma surgery. 2014;134(10):1381-6.

77. Larsen P, Lund H, Laessoe U, Graven-Nielsen T, Rasmussen S. Restrictions in Quality of Life After Intramedullary Nailing of Tibial Shaft Fracture: A Retrospective Follow-up Study of 223 Cases. Journal of orthopaedic trauma. 2014;28(9):507-12.
78. Tahririan MA, Ziaei E, Osanloo R. Significance of the position of the proximal tip of the tibial nail: An important factor related to anterior knee pain. Adv Biomed Res. 2014;3:119.
79. Sanders RW, DiPasquale TG, Jordan CJ, Arrington JA, Sagi HC. Semiextended intramedullary nailing of the tibia using a suprapatellar approach: radiographic results and clinical outcomes at a minimum of 12 months follow-up. Journal of orthopaedic trauma. 2014;28(5):245-55.
80. Ryan SP, Steen B, Tornetta P, 3rd. Semi-extended nailing of metaphyseal tibia fractures: alignment and incidence of postoperative knee pain. J Orthop Trauma. 2014;28(5):263-9.
81. Jones M, Parry M, Whitehouse M, Mitchell S. Radiologic outcome and patient-reported function after intramedullary nailing: a comparison of the retropatellar and infrapatellar approach. J Orthop Trauma. 2014;28(5):256-62.
82. Chen CY, Lin KC, Yang SW, Tarng YW, Hsu CJ, Renn JH. Influence of nail prominence and insertion point on anterior knee pain after tibial intramedullary nailing. Orthopedics. 2014;37(3):e221-5.
83. Moreschini O, Petrucci V, Cannata R. Insertion of distal locking screws of tibial intramedullary nails: A comparison between the free-hand technique and the SURESHOT™ Distal Targeting System. Injury. 2013;45(2):405-7.
84. Mir HR, Marinescu RC, Janda H, Russell TA. Biomechanical Effects of the Nail Entry Zone and Anterior Cortical Bone Loss on the Proximal Tibia. Journal of orthopaedic trauma. 2013;27(1):34-41.
85. Khan I, Javed S, Khan GN, Aziz A. Outcome of intramedullary interlocking SIGN nail in tibial diaphyseal fracture. Journal of the College of Physicians and Surgeons--Pakistan. 2013;23(3):203-7.
86. Rothberg DL, Daubs GM, Horwitz DS, Kubiak EN. One-year postoperative knee pain in patients with semi-extended tibial nailing versus control group. Orthopedics. 2013;36(5):e548-53.
87. Jankovic A, Korac Z, Bozic NB, Stedul I. Influence of knee flexion and atraumatic mobilisation of infrapatellar fat pad on incidence and severity of anterior knee pain after tibial nailing. Injury. 2013;44 Suppl 3:S33-9.
88. Gaines RJ, Rockwood J, Garland J, Ellingson C, Demaio M. Comparison of insertional trauma between suprapatellar and infrapatellar portals for tibial nailing. Orthopedics. 2013;36(9):e1155-8.
89. Bible JE, Choxi AA, Dhulipala S, Evans JM, Mir HR. Quantification of anterior cortical bone removal and intermeniscal ligament damage at the tibial nail entry zone using parapatellar and retropatellar approaches. J Orthop Trauma. 2013;27(8):437-41.
90. Aksahin E, Karasoy I, Hapa O, Dogan Ö, Duran S, Yüksel HY, et al. Does the change in the mass of parapatellar muscle influence the patellofemoral kinematics in the sagittal plane following the surgical treatment of tibial shaft fractures? Injury. 2013;44:S26.
91. Vaseenon T, Luevitoonvechkij S, Akkaraatimart W, Laohapoonrungsee A. Accurate Entry Point for Tibial Nailing with SIGN Nail in Asians: A Cadaveric Study. Journal of trauma and treatment. 2012;2012.
92. Theriault B, Turgeon AF, Pelet S. Functional Impact of Tibial Malrotation Following Intramedullary Nailing of Tibial Shaft Fractures. Journal of bone and joint surgery American volume. 2012;94(22):2033-9.
93. Song SY, Chang HG, Byun JC, Kim TY. Anterior knee pain after tibial intramedullary nailing using a medial paratendinous approach. J Orthop Trauma. 2012;26(3):172-7.
94. Schemitsch EH, Bhandari M, Guyatt G, Sanders DW, Swiontkowski M, Tornetta P, et al. Prognostic factors for predicting outcomes after intramedullary nailing of the tibia. The Journal of bone and joint surgery American volume. 2012;94(19):1786-93.
95. Attal R, Hansen M, Kirjavainen M, Bail H, Hammer TO, Rosenberger R, et al. A multicentre case series of tibia fractures treated with the Expert Tibia Nail (ETN). Archives of orthopaedic and trauma surgery. 2012;132(7):975-84.

96. Leliveld MS, Verhofstad MHJ. Injury to the infrapatellar branch of the saphenous nerve, a possible cause for anterior knee pain after tibial nailing? Injury. 2012;43(6):779-83.
97. Herren C, Graf M. The use of intraarticular semi-extended technique for treatment of tibial fractures. Injury. 2012;43:S7.
98. Cerqueira IS, Petersen PA, Júnior RM, Silva Jdos S, Reis P, Gaiarsa GP, et al. ANATOMICAL STUDY ON THE LATERAL SUPRAPATELLAR ACCESS ROUTE FOR LOCKED INTRAMEDULLARY NAILS IN TIBIAL FRACTURES. Revista brasileira de ortopedia. 2012;47(2):169-72.
99. Beltran MJ, Collinge CA, Patzkowski JC, Masini BD, Blease RE, Hsu JR. Intra-articular risks of suprapatellar nailing. American journal of orthopedics (Belle Mead, NJ). 2012;41(12):546-50.
100. Walker RM, Zdero R, McKee MD, Waddell JP, Schemitsch EH. Ideal tibial intramedullary nail insertion point varies with tibial rotation. Journal of orthopaedic trauma. 2011;25(12):726-30.
101. Ryan SP, Tornetta P, 3rd, Dielwart C, Kaye-Krall E. Knee pain correlates with union after tibial nailing. Journal of orthopaedic trauma. 2011;25(12):731-5.
102. Labronici PJ, Santos Pires RE, Franco JS, Alvachian Fernandes HJ, Dos Reis FB. Recommendations for avoiding knee pain after intramedullary nailing of tibial shaft fractures. Patient safety in surgery. 2011;5(1):31.
103. Freeman AL, Craig MR, Schmidt AH. Biomechanical comparison of tibial nail stability in a proximal third fracture: do screw quantity and locked, interlocking screws make a difference? Journal of orthopaedic trauma. 2011;25(6):333-9.
104. Forman JM, Urruela AM, Egol KA. The percutaneous use of a pointed reduction clamp during intramedullary nailing of distal third tibial shaft fractures. Acta orthopaedica Belgica. 2011;77(6):802-8.
105. Darabos N, Bajs ID, Rutić Z, Darabos A, Poljak D, Dobsa J. Nail position has an influence on anterior knee pain after tibial intramedullary nailing. Collegium antropologicum. 2011;35(3):873-7.
106. Manoj Kumar Chakraborty PT, Brijesh Sathian. Surgical Implant Generation Network (SIGN) Solid Intramedullary Interlocking Nail in the Lower Extremity: An Observational Study from Western Nepal. Journal of Clinical and Diagnostic Research. 2011;5(8):1614-7.
107. Sadeghpour A, Mansour R, Aghdam HA, Goldust M. Comparison of trans patellar approach and medial parapatellar tendon approach in tibial intramedullary nailing for treatment of tibial fractures. JPMA The Journal of the Pakistan Medical Association. 2011;61(6):530-3.
108. Weninger P, Tschabitscher M, Traxler H, Pfafl V, Hertz H. Influence of medial parapatellar nail insertion on alignment in proximal tibia fractures--special consideration of the fracture level. The Journal of trauma. 2010;68(4):975-9.
109. Kubiak EN, Widmer BJ, Horwitz DS. Extra-articular technique for semiextended tibial nailing. Journal of orthopaedic trauma. 2010;24(11):704-8.
110. Gelbke MK, Coombs D, Powell S, DiPasquale TG. Suprapatellar versus infra-patellar intramedullary nail insertion of the tibia: a cadaveric model for comparison of patellofemoral contact pressures and forces. J Orthop Trauma. 2010;24(11):665-71.
111. Eastman JG, Tseng SS, Lee MA, Yoo BJ. The retropatellar portal as an alternative site for tibial nail insertion: a cadaveric study. J Orthop Trauma. 2010;24(11):659-64.
112. Eastman J, Tseng S, Lo E, Li CS, Yoo B, Lee M. Retropatellar technique for intramedullary nailing of proximal tibia fractures: A cadaveric assessment. Journal of orthopaedic trauma. 2010;24(11):672-6.
113. Weninger P, Schultz A, Traxler H, Firbas W, Hertz H. Anatomical assessment of the Hoffa fat pad during insertion of a tibial intramedullary nail--comparison of three surgical approaches. The Journal of trauma. 2009;66(4):1140-5.
114. Weil YA, Gardner MJ, Boraiah S, Helfet DL, Lorich DG. Anterior knee pain following the lateral parapatellar approach for tibial nailing. Archives of orthopaedic and trauma surgery. 2009;129(6):773-7.
115. Uzümcügil O, Doğan A, Yalçinkaya M, Kabukçuoğlu YS. The relationship between anterior knee pain occurring after tibial intramedullary nailing and the localization of the nail in the proximal tibia. Acta orthopaedica et traumatologica turcica. 2009;43(5):386-9.

116. Lefaivre KA, Guy P, Chan H, Blachut PA. Long-term follow-up of tibial shaft fractures treated with intramedullary nailing. Journal of orthopaedic trauma. 2008;22(8):525-9.
117. Fanian H, Dehghani M. Anterior knee pain after unreamed intramedullary nailing of the tibia. Journal of research in medical sciences. 2008;13(5):260-3.
118. Bhandari M, Guyatt G, Tornetta P, Schemitsch EH, Swiontkowski M, Sanders D, et al. Randomized trial of reamed and unreamed intramedullary nailing of tibial shaft fractures. Journal of bone and joint surgery American volume. 2008;90A(12):2567-78.
119. Väistö O, Toivanen J, Kannus P, Järvinen M. Anterior knee pain after intramedullary nailing of fractures of the tibial shaft: An eight-year follow-up of a prospective, randomized study comparing two different nail-insertion techniques. Journal of Trauma - Injury, Infection and Critical Care. 2008;64(6):1511-6.
120. Giri SK, Adhikari BR, Gurung GB, Rc D, Bajracharya AR, Khatri K. Mini-open reduction and intramedullary interlocking nailing of fracture shaft of tibia without an image intensifier. Nepal Medical College journal : NMCJ. 2008;10(2):123-5.
121. Väistö O, Toivanen J, Kannus P, Järvinen M. Anterior knee pain and thigh muscle strength after intramedullary nailing of a tibial shaft fracture: an 8-year follow-up of 28 consecutive cases. J Orthop Trauma. 2007;21(3):165-71.
122. Cartwright-Terry M, Snow M, Nalwad H. The Severity and Prediction of Anterior Knee Pain Post Tibial Nail Insertion. Journal of orthopaedic trauma. 2007;21(6):381-5.
123. Vidyadhara S, K. Rao S. Prospective study of the clinico-radiological outcome of interlocked nailing in proximal third tibial shaft fractures. Injury. 2006;37(6):536-42.
124. Nork SE, Barei DP, Schildhauer TA, Agel J, Holt SK, Schrick JL, et al. Intramedullary Nailing of Proximal Quarter Tibial Fractures. Journal of orthopaedic trauma. 2006;20(8):523-8.
125. Bhattacharyya T, Seng K, Nassif NA, Freedman I. Knee pain after tibial nailing : The role of nail prominence. Clinical orthopaedics and related research. 2006;449(449):303-7.
126. Babis GC, Benetos IS, Karachalios T, Soucacos PN. Eight years' clinical experience with the Orthofix® tibial nailing system in the treatment of tibial shaft fractures. Injury. 2006;38(2):227-34.
127. Nork SE, Schwartz AK, Agel J, Holt SK, Schrick JL, Winquist RA. Intramedullary Nailing of Distal Metaphyseal Tibial Fractures. Journal of bone and joint surgery American volume. 2005;87(6):1213-21.
128. Fan C-Y, Chiang C-C, Chuang T-Y, Chiu F-Y, Chen T-H. Interlocking nails for displaced metaphyseal fractures of the distal tibia. Injury. 2005;36(5):669-74.
129. Audige L, Griffin D, Bhandari M, Kellam J, RÜEdi TP. Path analysis of factors for delayed healing and nonunion in 416 operatively treated tibial shaft fractures. Clinical orthopaedics and related research. 2005;438(438):221-32.
130. Al Hussainy HAJ, Deeb A, Choudhary AK. Anterior knee pain following intramedullary nailing of tibial shaft fractures: does bony portal point in the sagittal plane affect the outcome? European journal of orthopaedic surgery & traumatology. 2005;15(2):113-7.
131. Väistö O, Toivanen J, Paakkala T, Järvelä T, Kannus P, Järvinen M. Anterior knee pain after intramedullary nailing of a tibial shaft fracture: an ultrasound study of the patellar tendons of 36 patients. Journal of orthopaedic trauma. 2005;19(5):311-6.
132. Väistö O, Toivanen J, Kannus P, Järvinen M. Anterior Knee Pain and Thigh Muscle Strength After Intramedullary Nailing of Tibial Shaft Fractures: A Report of 40 Consecutive Cases. Journal of orthopaedic trauma. 2004;18(1):18-23.
133. Puloski S, Romano C, Buckley R, Powell J. Rotational Malalignment of the Tibia Following Reamed Intramedullary Nail Fixation. Journal of orthopaedic trauma. 2004;18(7):397-402.
134. Laflamme GY, Heimlich D, Stephen D, Kreder HJ, Whyne CM. Proximal Tibial Fracture Stability With Intramedullary Nail Fixation Using Oblique Interlocking Screws. Journal of orthopaedic trauma. 2003;17(7):496-502.
135. Samuelson MA, McPherson EJ, Norris L. Anatomic Assessment of the Proper Insertion Site for a Tibial Intramedullary Nail. Journal of orthopaedic trauma. 2002;16(1):23-5.
136. Kahn KM, Beals RK. Malrotation after locked intramedullary tibial nailing: three case reports and review of the literature. The Journal of trauma. 2002;53(3):549-52.

137. Gorczyca JT, McKale J, Pugh K, Pienkowski D. Modified tibial nails for treating distal tibia fractures. Journal of orthopaedic trauma. 2002;16(1):18-22.
138. Dogra AS, Ruiz AL, Marsh DR. Late outcome of isolated tibial fractures treated by intramedullary nailing: the correlation between disease-specific and generic outcome measures. J Orthop Trauma. 2002;16(4):245-9.
139. Toivanen JA, Vaisto O, Kannus P, Latvala K, Honkonen SE, Jarvinen MJ. Anterior knee pain after intramedullary nailing of fractures of the tibial shaft. A prospective, randomized study comparing two different nail-insertion techniques. The Journal of bone and joint surgery American volume. 2002;84-a(4):580-5.
140. Ricci WM, O'Boyle M, Borrelli J, Bellabarba C, Sanders R. Fractures of the proximal third of the tibial shaft treated with intramedullary nails and blocking screws. Journal of orthopaedic trauma. 2001;15(4):264-70.
141. Nyland J, Bealle DP, Kaufer H, Johnson DL. Long-term quadriceps femoris functional deficits following intramedullary nailing of isolated tibial fractures. International Orthopaedics. 2001;24(6):342-6.
142. McConnell T, Tornetta P, 3rd, Tilzey J, Casey D. Tibial portal placement: the radiographic correlate of the anatomic safe zone. Journal of orthopaedic trauma. 2001;15(3):207-9.
143. Lembcke O, Rüter A, Beck A. The nail-insertion point in unreamed tibial nailing and its influence on the axial malalignment in proximal tibial fractures. Archives of orthopaedic and trauma surgery. 2001;121(4):197-200.
144. Tyllianakis M, Megas P, Giannikas D, Lambiris E. Interlocking intramedullary nailing in distal tibial fractures. Orthopedics. 2000;23(8):805-8.
145. Karachalios T, Babis G, Tsarouchas J, Sapkas G, Pantazopoulos T. The clinical performance of a small diameter tibial nailing system with a mechanical distal aiming device. Injury. 2000;31(6):451-9.
146. Hernigou P, Cohen D. Proximal entry for intramedullary nailing of the tibia. The risk of unrecognised articular damage. The Journal of bone and joint surgery British volume. 2000;82(1):33-41.
147. Finkemeier CG, Schmidt AH, Kyle RF, Templeman DC, Varecka TF. A prospective, randomized study of intramedullary nails inserted with and without reaming for the treatment of open and closed fractures of the tibial shaft. Journal of orthopaedic trauma. 2000;14(3):187-93.
148. Dogra AS, Ruiz AL, Thompson NS, Nolan PC. Dia-metaphyseal distal tibial fractures--treatment with a shortened intramedullary nail: a review of 15 cases. Injury. 2000;31(10):799-804.
149. Krettek C, Stephan C, Schandelmaier P, Richter M, Pape HC, Miclau T. The use of Poller screws as blocking screws in stabilising tibial fractures treated with small diameter intramedullary nails. The Journal of bone and joint surgery British volume. 1999;81(6):963-8.
150. Tornetta 3rd P, Riina J, Geller J, Purban W. Intraarticular anatomic risks of tibial nailing. Journal of orthopaedic trauma. 1999;13(4):247-51.
151. Moed BR, Subramanian S, van Holsbeeck M, Watson JT, Cramer KE, Karges DE, et al. Ultrasound for the early diagnosis of tibial fracture healing after static interlocked nailing without reaming: clinical results. Journal of orthopaedic trauma. 1998;12(3):206-13.
152. Lovell ME, Sharma S, Allcock S, Hardy SK. Insertion site for intramedullary tibial nails, and its relationship to anterior knee pain. The knee. 1998;5(4):253-4.
153. Krettek C, Könemann B, Farouk O, Miclau T, Kromm A, Tscherne H. Experimental study of distal interlocking of a solid tibial nail: radiation-independent distal aiming device (DAD) versus freehand technique (FHT). Journal of orthopaedic trauma. 1998;12(6):373-8.
154. Devitt AT, Coughlan KA, Ward T, McCormack D, Mulcahy D, Felle P, et al. Patellofemoral contact forces and pressures during intramedullary tibial nailing. International orthopaedics. 1998;22(2):92-6.
155. Hallam P, Ferris B. Patellar tendon split or parapatellar approach for tibial nailing? Does it affect the patellar height? Knee. 1998;5(3):203-4.
156. Tornetta P, 3rd, French BG. Compartment pressures during nonreamed tibial nailing without traction. Journal of orthopaedic trauma. 1997;11(1):24-7.
157. Keating JF, Orfaly R, O'Brien PJ. Knee pain after tibial nailing. Journal of orthopaedic trauma. 1997;11(1):10-3.
158. Court-Brown CM, Gustilo T, Shaw AD. Knee pain after intramedullary tibial nailing: its incidence, etiology, and outcome. J Orthop Trauma. 1997;11(2):103-5.
159. Buehler KC, Green J, Woll TS, Duwelius PJ. A technique for intramedullary nailing of proximal third tibia fractures. Journal of orthopaedic trauma. 1997;11(3):218-23.

160. Blachut PA, O'Brien PJ, Meek RN, Broekhuyse HM. Interlocking intramedullary nailing with and without reaming for the treatment of closed fractures of the tibial shaft. A prospective, randomized study. The Journal of bone and joint surgery American volume. 1997;79(5):640-6.
161. Kneifel T, Buckley R. A comparison of one versus two distal locking screws in tibial fractures treated with unreamed tibial nails: a prospective randomized clinical trial. Injury. 1996;27(4):271-3.
162. Court-Brown CM, Will E, Christie J, McQueen MM. Reamed or unreamed nailing for closed tibial fractures. A prospective study in Tscherne C1 fractures. The Journal of bone and joint surgery British volume. 1996;78(4):580-3.
163. Boenisch UW, de Boer PG, Journeaux SF. Unreamed intramedullary tibial nailing--fatigue of locking bolts. Injury. 1996;27(4):265-70.
164. Tornetta Iii P, Collins E. Semiextended position for intramedullary nailing of the proximal tibia. Clinical orthopaedics and related research. 1996(328):185-9.
165. Orfaly R, Keating JE, O'Brien PJ. Knee pain after tibial nailing: does the entry point matter? The Journal of bone and joint surgery British volume. 1995;77(6):976-7.
166. Lang GJ, Cohen BE, Bosse MJ, Kellam JF. Proximal third tibial shaft fractures. Should they be nailed? Clinical Orthopaedics and Related Research. 1995(315):64-74.
167. Gregory P, Sanders R. The treatment of closed, unstable tibial shaft fractures with unreamed interlocking nails. Clinical orthopaedics and related research. 1995(315):48-55.
168. Freedman EL, Johnson EE. Radiographic analysis of tibial fracture malalignment following intramedullary nailing. Clinical Orthopaedics and Related Research. 1995(315):25-33.
169. Anglen JO, Blue JM. A comparison of reamed and unreamed nailing of the tibia. The Journal of trauma. 1995;39(2):351-5.
170. O'Dwyer KJ, Chakravarty RD, Esler CN. Intramedullary nailing technique and its effect on union rates of tibial shaft fractures. Injury. 1994;25(7):461-4.
171. Habernek H, Kwasny O, Schmid L, Ortner F. Complications of interlocking nailing for lower leg fractures: a 3-year follow up of 102 cases. The Journal of trauma. 1992;33(6):863-9.
172. Koval KJ, Clapper MF, Brumback RJ, Ellison PS, Jr., Poka A, Bathon GH, et al. Complications of reamed intramedullary nailing of the tibia. Journal of orthopaedic trauma. 1991;5(2):184-9.
173. Carr J, Sobba D, Bear L. Biomechanics of Rigid Tibial Nail Insertion Sites. Journal of orthopaedic trauma. 1991;5(2):238.
174. Court-Brown CM, Christie J, McQueen MM. Closed intramedullary tibial nailing. Its use in closed and type I open fractures. The Journal of bone and joint surgery British volume. 1990;72(4):605-11.
175. Alho A, Ekeland A, Strømsøe K, Follerås G, Thoresen BO. Locked intramedullary nailing for displaced tibial shaft fractures. The Journal of bone and joint surgery British volume. 1990;72(5):805-9.
176. Ekeland A, Thoresen BO, Alho A, Strömsöe K, Follerås G, Haukebø A. Interlocking intramedullary nailing in the treatment of tibial fractures. A report of 45 cases. Clinical orthopaedics and related research. 1988(231):205-15.
177. Bone LB, Johnson KD. Treatment of tibial fractures by reaming and intramedullary nailing. The Journal of bone and joint surgery American volume. 1986;68(6):877-87.
178. Donald G, Seligson D. Treatment of tibial shaft fractures by percutaneous Küntscher nailing. Technical difficulties and a review of 50 consecutive cases. Clinical orthopaedics and related research. 1983(178):64-73.
179. Hamza KN, Dunkerley GE, Murray CM. Fractures of the tibia. A report on fifty patients treated by intramedullary nailing. The Journal of bone and joint surgery British volume. 1971;53(4):696-700.
